# Can a More Variable Species Win Interspecific Competition?

**DOI:** 10.1007/s10441-021-09408-0

**Published:** 2021-02-12

**Authors:** Janusz Uchmański

**Affiliations:** 1 Cardinal Stefan Wyszyński University, Wóycickiego 1/3, 01-938 Warsaw, Poland; 2 Tyumen State University, 10 Semakov St., Tyumen, Russia 625003

**Keywords:** Individual-based model, Individual variation, Competition, Extinction time

## Abstract

An individual-based approach is used to describe population dynamics. Two kinds of models have been constructed with different distributions illustrating individual variability. In both models, the growth rate of an individual and its final body weight at the end of the growth period, which determines the number of offspring, are functions of the amount of resources assimilated by an individual. In the model with a symmetric distribution, the half saturation constant in the Michaelis–Menten function describing the relationship between the growth of individuals and the amount of resources has a normal distribution. In the model with an asymmetric distribution, resources are not equally partitioned among individuals. The individual who acquired more resources in the past, will acquire more resources in the future. A single population comprising identical individuals has a very short extinction time. If individuals differ in the amount of food assimilated, this time significantly increases irrespectively of the type of model describing population dynamics. Individuals of two populations of competing species use common resources. For larger differences in individual variability, the more variable species will have a longer extinction time and will exclude less variable species. Both populations can also coexist when their variabilities are equal or even when they are slightly different, in the latter case under the condition of high variability of both species. These conclusions have a deterministic nature in the case of the model with the asymmetric distribution—repeated simulations give the same results. In the case of the model with the symmetric distribution, these conclusions are of a statistical nature—if we repeat the simulation many times, then the more variable species will have a longer extinction time more frequently, but some results will happen (although less often) when the less variable species has a longer extinction time. Additionally, in the model with the asymmetric distribution, the result of competition will depend on the way of the introduction of variability into the model. If the higher variability is due to an increase in the proportion of individuals with a low assimilation of resources, it can produce a longer extinction time of the less variable species.

## Introduction

The competitive exclusion principle was an important and intensively discussed question in theoretical ecology of the sixties, seventies, and eighties. At that time classical Volterrian models in their original form describing the density dynamics of populations and communities (Volterra 1931) were in common use. In the case of two competing species, they show that both species can persistently coexist only in the situation when intraspecific competition is stronger than interspecific competition. In other cases, the density of one species reaches zero, while the density of the second species asymptotically approaches nonzero value. Parameter values of the excluded species indicate that it is more than other species vulnerable to interspecific competition. When both species are equal competitors, the initial densities decide who will win the interspecific competition (this species which initially had greater density).

In the classical form of Volterra model of the dynamics of two competing species resources are not explicit. Competition depends on the terms proportional to the product of the densities of both competing species. Competitively excluded is the species with a higher absolute value of the coefficient preceding this product (both these terms are placed with negative signs in the model equations, as competition reduces the growth rate of each competitor, although not equally).

The model of the population dynamics of two competing species proposed by Tilman (1977, 1982) is developed on a different basis. Resources are explicit in this model. Their dynamics is described by an additional equation. The two remaining equations describe, as earlier, the dynamics of the density of two competing species. Competition is for the same resources (this is the only form of information which these competitors have about one another), and the rate of increase in their densities is an increasing Michaelis–Menten function of the amount of resources. The winner of competition is the species with the lowest half saturation constant.

One can infer from this kind of models that the coexistence of species is possible only when the interspecific competition in relation to the intraspecific one is ecologically unimportant factor. However, Armstrong and McGehee (1976, 1980) argued that the above holds only for Volterrian model of competition in its original form which is a linear one. If we assume some nonlinear functions of densities on the right hand side of the model’s equation and additionally allow the densities to fluctuate, for instance, in the form of a limit cycle, then we obtain the coexistence of two (or even more (Zicarelli 1975)) competing species (Koch 1974; McGehee and Armstrong 1977). Abrams (1984) showed that temporal variation in resource assimilation—especially when there is a strong negative correlation between the assimilation rates of different species—promotes the coexistence of competing species. Presence of a predator can also positively influence competitors coexistence. Teramoto et al. (1979) and Messia et al. (1984) illustrated this with models in which a predator species exploits two prey species. The prey species are competitors. However, the predator species has the ability to switch on this prey which is more abundant. This mechanism enables the coexistence of the competitors. In the absence of the predator, the density of one of the competitor species reaches zero.

When we are looking at the ecological literature of that time, especially interesting from our point of view is the paper of Begon and Wall (1987). They have used the model of Hassell and Comins (1976) describing the dynamics of two competing species to answer the question whether species variability can facilitate competitors coexistence. Classical ecological models with population density as the state variable are using parameters which are the average characteristics of interacting species. That’s why it is not easy to introduce individual variability into them. One possibility is to apply so-called structured population models (Ebenman and Persson 1988). Begon and Wall divided the total densities of both competing species into classes with different carrying capacities (intraspecific competition ability) and different interspecific competitive ability. When there is an individual variability in the parameters describing intra- and interspecific competition, both species coexist. This effect has been observed only for sufficiently high degree of variability to ensure that for the inferior competitors the class with the highest interspecific competition ability was superior than the class of lowest interspecific competition ability of the superior competitor. Without individual variability and parameter values equal to averages exclusion of inferior species was observed.

Later the ecological discussion on the dynamics of competing species has been shifted to solving different problems. Instead of considering how to secure the persistence of identical or strongly interacting competitors, ecologists started to think how different should be the competing species to coexist (May 1973, 1981). Species don’t compete for one resource. Let us assume that there are different kinds of resources and they can be described by some continuous variable. Each species has its own function of resource utilization in the form of Gaussian distribution. So-called principle of limiting similarity (Abrams 1983) indicates the minimal distance between the maxima of these functions of different competitors which ensure their coexistence.

In the nineties, all these theoretical problems almost totally disappeared from the ecological literature and only recently Clark (2010) and Violle et al. (2012) recalled the problem of the coexistence of species and the role of intraspecific variability in the dynamics of competing species. Several models have appeared in the last decade. Lichstein et al. 2007 presented a stochastic model for two competing plant species: all adults are identical, they produce the same number of seeds, individual variability exists only among juveniles, which differ in their competitive ability. There is no explicit space in the model. Proportion of places occupied by adults (equal to adult density) was calculated in the model. It was assumed that empty places are occupied by the best juvenile. When juveniles of both species have identical distributions of competitive ability, the competitive exclusion is random with probability 0.5. For different distributions of competitive ability, the conditions for exclusion of one species and for coexistence of both species were formulated. The results of the model strongly depend on the density of competing juveniles. Another plant model was analyzed by Hart et al. (2016).They adopted the model originally developed by Beverton and Holt (1957) for fish population dynamics. The model equations describe the dynamics of seed densities. Only the competition between seeds is assumed. The authors argue that contrary to common belief, the intraspecific variation can reduce the likelihood of competing species coexistence. It is due to the nonlinear relationships between the dynamics of population and demographic rates included in the model and because of demographic stochasticity.

The opposite results were obtained by Menden-Deuer and Rowlett (2014) in the model for the dynamics of competing microorganisms. The model consists of differential equations for the dynamics of competing populations, but the rate of density increase for each population depends on the cumulative payoff of all individuals of this population playing a competitive game against other individuals of all species. The individual variability in strategies ensure the coexistence of competing species. As computer simulations show, this result is valid for communities consisting up to 100 species. Competitive exclusion was observed only in the case of very small populations. Feniova et al. (2013) used a numerical scheme with parameter values taken from laboratory experiments on the increase in numbers of *Simocephalus vetulus* and *Ceriodaphnia quadrangular* clones, the traits of which differed. She proved that even the less competitive species could exclude the stronger competitor on the condition that the first occurred in the form of several different clones, and the second only in the form of one nondiversified clone. Moreover, the authors indicate the effect of temperature on the expression of individual variation—it increases with increasing temperature.

The aim of this paper is an analysis of the effect of individual variability on the outcome of interspecific competition. Unlike in the models previously mentioned, this paper will consider a pure, relatively general individual-based model of two competing species describing growth and reproduction of individuals that differ in important traits with respect to the utilization of resources. Resource dynamics will be explicit in the model. Competition will be of global type and its outcome—uneven resource partitioning—will influence the rate of growth of individuals.

Individual variability is obvious for ecologists, but it is absent from classical mathematical models of ecological systems. We know quite a lot about the variation of body weight in even-aged populations of plants and animals (Uchmański 1985; Huston 1994). Frequency distributions of such weights are positively skewed, and their skewness increases with, for example, population density, or deteriorating food conditions. The cause of the positive skewness of weight distribution in even-aged populations is attributed to intraspecific competition. Individual body weight is a cumulative variable. Its value in the period of growth before the age of reproduction is an effect of the summation of the excess of energy intake from the environment over the general cost of life, which can be measured as the rate of respiration by an organism (Majkowski and Uchmański 1980). Competition means uneven resource partitioning among competitors (Łomnicki 1988). If this is food, then food assimilation by different members of an even-aged population will differ. As a result, the body weights will have some distribution. The shape of this distribution, its positive skewness, is a result of another attribute of competition as expressed by the following statement: who acquired more food in the past, will acquire even more of it in the future (Uchmański 1987). Therefore, if we arrange individuals in even-aged population according to increasing body weight, then we will be able to assign a certain rate of food assimilation to each of them, and later to describe this assimilation as a function of body weight. It turns out that to get a positive skewness of the body weights in even-aged populations this function should be non-concave (thus linear or convex), and dependent in a specific way on the amount of food available in the environment (Uchmański 1985; Uchmański and Dgebuadze 1990).

Individual variability defined in this way is important to the dynamics of a single population. This problem was analyzed for the first time by Łomnicki (1978). However, he used the classical model of a single population, thus he did not refer to any pattern considering the traits of individuals and interactions among them known from experiments and observations. Individual-based models, directly incorporating the pattern of growth of individuals and resource partitioning among them as it was presented above, yield quite different properties of population dynamics from these which we know from classical models. General properties of population dynamics in such models were analyzed by Uchmański (2000a) and Grimm and Uchmański (2002), and the effect of different ways of resource partition on population dynamics by Uchmański (2000b).

The analysis of earlier papers dealing with interspecific competition presented above shows that when individual variability was included into the model, the distribution illustrating it was symmetric. However, the deeper ecological analysis of individual variability produced during interactions between individuals, as we can see from two above paragraphs, indicates that individual variability should be described rather by positively skewed distributions.

This is the reason why two versions of the model will be considered in this paper: with a symmetric distribution describing individual variability and with an asymmetric one. The model with a symmetric distribution assuming random individual variability will be treated as null-model for the model with an asymmetric one. In this last case, it is assumed that individual variability is an effect of competition. The aim of the model with an asymmetric distribution is to extend the above presented scheme describing resource partitioning resulting from intraspecific competition between individuals, which so far was used to develop models of single populations, to a two-species case, and to examine the significance of this type of individual variability to the results of interspecific competition.

## Random Individual Variability

### A Single Population

The model describes the population dynamics of animals with nonoverlapping generations and the dynamics of resources available to them. The lifecycle of individuals starts at the beginning of the season. They grow over the season and reproduce at the end of the season, then they die. Juveniles overwinter and start growing at the beginning of the next season. The individuals represent a parthenogenetic species.

The growth rate of an individual is assessed as the difference between the rate of resource assimilation and the rate with which these assimilated resources are used for living costs. The rate of resource assimilation *A* and living costs as measured by the rate of respiration *R* are power functions of body weight *w* (Duncan and Klekowski 1975):

1 $$A = a_{1} w^{b_1 }$$

2 $$R = a_{2} w^{b_2}$$

where *a*_1_, *a*_2_, *b*_1_ and b_2_ are parameters. This gives the following equation of individual growth (Majkowski and Uchmański 1980):

3 $$dw/dt \, = \, a_{{1}} w^{b_1 } - a_{{2}} w^{b_2}$$

The rate of assimilation depends on the amount of available food resources. Let us describe this relationship in the same way as Tilman (1977, 1982) did it, by using the Michaelis–Menten curve applied for filtering organisms (Murray 1989):

4 $$a_{{1}} = \, a_{{{1},max}} V/\left( {H + V} \right)$$

where *V* is the amount of resources available in the environment, *H* is the half saturation constant, that is, the amount of resources at which the rate of their assimilation reaches half of the maximum value and *a*_1,*max*_ is the maximal value of parameter *a*_1_ reached when *V* = ∞.

The greatest weight *w*_*max*_ (at successive time steps of the simulation and at the end of growth) has a hypothetical individual who is growing under conditions *V* = ∞:

5 $$dw_{{\max }} /dt{\mkern 1mu} = {\mkern 1mu} a_{{1,\max }} w_{{\max }}^{{b_{1} }} - a_{2} w_{{\max }}^{{b_{2} }}$$

The maximum final weight *w*_*max*_^*end*^ of an individual, asymptotically reached when assimilation is equal to respiration, for the growth described by Eq. (5) is

6 $$w_{{\max }}^{{end}} = {\mkern 1mu} \left( {a_{{1,\max }} /a_{2} } \right)^{{1/{\kern 1pt} (b_2 - b_1)}}$$

An individual growing under condition when *V* < ∞, after the end of growth will reach the weight *w*_*end*_ < *w*_*max*_^*end*^. The number of juveniles produced by an individual after the end of growth is proportional to the difference between its final weight and some threshold weight:

7 $$z\; = \;\left\{ {\begin{array}{*{20}c} {round \, (c(w_{end} - w_{fak} w_{\max }^{end} )} & {{\text{for}}\;w_{end} > w_{fak} w_{\max }^{end} } \\ 0 & {{\text{for}}\;w_{end} \le w_{fak} w_{\max }^{end} } \\ \end{array} } \right\}$$

where *c* is the parameter describing the intensity of juvenile production, and *w*_*fak*_ (0 < *w*_*fak*_ < 1) says what part of the maximum end weight *w*_*max*_^*end*^ given by Eq. (6) is the threshold weight which allows the calculation of juvenile production by an individual. Individuals with body weights lower than or equal to the threshold weight die before producing progeny. The function *round* rounds a real number to the nearest integer, as the number of juveniles can be only a natural number. The initial weights of juveniles of each individual are drawn from the normal distribution with a mean value *w*_0*, mean*_ and variance *w*_0,variance_, but only from the interval [*w*_0*,min*_, *w*_0*,max*_].

The number of individuals in the population *N*_*t*+1_ at time step *t* + 1 conforms to the following equation

8 $$N_{{_{{t + 1}} }} = \Sigma z_{i}$$

where the summation is done over all individuals present in the population at time step *t*. This was combined with the equation describing the resource dynamics

9 $$V_{{t + 1}} = V_{t} - \Sigma A_{i} + g$$

where *V*_*t*+1_ and *V*_*t*_ are resources in two subsequent time steps, *A*_*i*_ is the resource assimilation by the *i*-th individual and *g* is the constant amount of resources added at each time step. This assumption made here for the sake of simplicity means that resources not consumed by individuals in the population will linearly increase. In Eq. (9) the summation is also over all individuals present in the population at time step *t*.

At the initial time instant, the population consisted of *N*_0_ individuals, and they had *Vo* available resources. Their initial weights are taken from the normal distribution with the properties noted above. The basic simulation step was that used for the calculation of the number of individuals in the population of successive generations. However, within each generation, the equations describing the growth of individuals and the resource equation were solved by using the Euler method in 80 smaller time steps. This number of smaller steps allowed a good enough fitting of the numerical solutions to the analytical solutions of the growth Eq. (5) for an individual with the maximum weight. Weight increases at each smaller time step were calculated in the model with reference to the actual amount of resources available to individuals. After the end of growth, the number of juveniles for each individual was calculated, their initial weights were assessed, and the amount of food was calculated with respect to its utilization and supplementation. This allowed for the same calculations at successive time steps. The simulation was stopped when *N*_*t*+1_ = 0 or *V*_*t*+1_ < 0.

In an even-aged population, individuals differ in the values of the parameter *H*. For each individual, it is drawn from a normal distribution with a mean value *H*_*mean*_ and variance *H*_*variance*_. To avoid meaningless values of this parameter, it is assumed that the drawn values of *H* should fulfill the condition

10 $$H_{min} \le H \le H_{max}$$

where *H*_*min*_ and *H*_*max*_ are parameters. At a constant level of resources *V*, the individual with a lover value of the parameter *H* will have a higher rate of consumption, attain a greater final weight, and produce more juveniles.

### Two Competing Species

Individuals of different species “feel” their presence as they use the common food supply. The equation describing resource dynamics is of the form

11 $$V_{{t + 1}} = V_{t} - \Sigma {A_{i}^{1} } - \Sigma {A_{i}^{2} } + g$$

where the first sum is the joint total assimilation by individuals of species 1 and the second of species 2.

In the simulations, all parameters describing different species were the same, except for those describing individual variability in the values of parameter *H* (Table 1). However, the mean, the highest and lowest values of this parameter were the same for both species (*H*_*mean*_^1^ = *H*_*mean*_^2^, *H*_*max*_^1^ = *H*_*max*_^2^, *H*_*min*_^1^ = *H*_*min*_^2^). The difference in individual variation between the species means that these species differed in the variances of the distributions from which the values of the parameter *H* were drawn (*H*_*variance*_^1^ ≠ *H*_*variance*_^2^).

**Table 1:** Standard values of the model parameters used in the simulation of competition between two species

	Parameter	Value
Growth equation parameters	*a*_1,*max*_	0.11
–	*a*_2_	0.03
–	*b*_1_	0.7
–	*b*_2_	0.9
Parameters of initial weight distribution	*w*_0,*min*_	14
–	*w*_0,*max*_	26
–	*w*_0,*mean*_	20
–	*w*_*0,variance*_	5
Parameters of half saturation constant distribution	*H*_*mean*_	500·10^3^
–	*H*_*variance*_	50 × 10^3^ to 700 × 10^3^
–	*H*_*min*_	100 × 10^3^
–	*H*_*max*_	1000 × 10^3^
Threshold for reproduction	*w*_*fak*_	0.65
Initial number	*N*_0_	5
Initial resources	*V*_0_	6 × 10^6^
Increase of resources	*g*	2 × 10^6^

Indexes indicating species number are not shown with parameter symbols, as they differ only in the values of *H*_*variance*_. For *H*_*variance*_ only the range of its variance is shown, as the simulation outcomes are presented for different values of this parameter

### Results

#### A Single Population. Identical Individuals

**Fig. 1:**
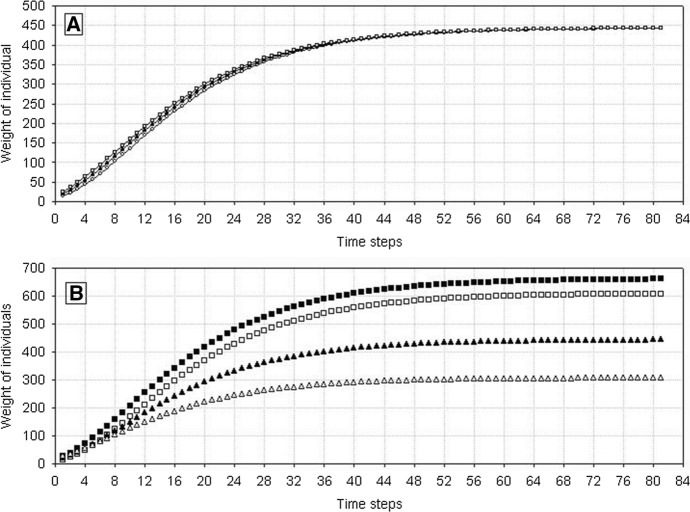
Growth curves of individuals [according to Eqs. (3) and (4)] in the constant resource condition *V* = 6 × 10^6^. **A** Growth of individuals with various initial weights, but with the same value of *H* = 500 × 10^3^. Empty squares—*w*_0_ = 26, filled triangles—*w*_0_ = 20, empty circles—*w*_0_ = 14. **B** Differences in the initial body weights are maintained, but individuals differ in the value of *H*. Empty squares—*H* = 100 × 10^3^, filled triangles—*H* = 500 × 10^3^, empty triangles—*H* = 1000 × 10^3^.^.^ The highest curve (filled quadrates) illustrates the growth of the heaviest possible individual growing in the condition when *V* = ∞ [Eq. (5)]

In a population comprising identical individuals, each of them will grow in the same way. As the initial weights of individuals were drawn from a normal distribution, thus at very early growth stages, their growth curves only slightly differed from each other, and these differences soon disappeared, so that at the end of growth all individuals had the same final weight (Fig. 1A). First, this happens because such is the nature of the balanced growth equation (Eq. 3) used in this model (Majkowski and Uchmański 1980), but first of all, because each individual responds in the same way to the amount of resources actually available in the environment.

**Fig. 2:**
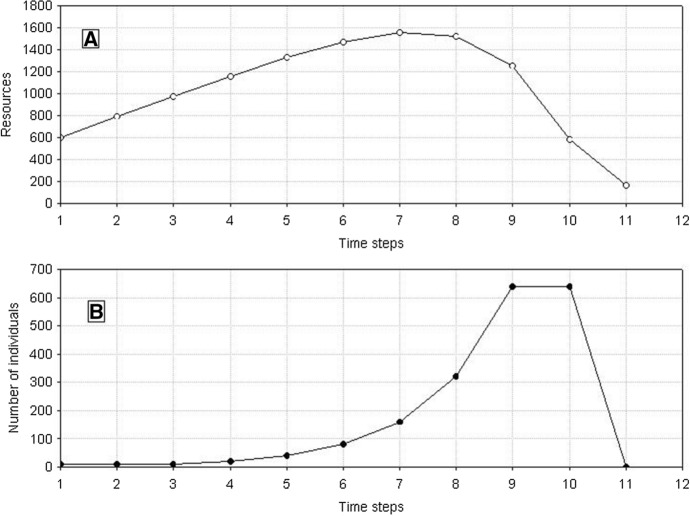
Number dynamics of a population composed of identical individuals. Standard parameter values. In simulations of the dynamics of a single population, the initial numbers were twice as high (*N*_0_ = 10) as in simulations of the competition between two species (see Table 1) to obtain similar initial conditions. All individuals have the same value of the parameter *H* = 500 × 10^3^. **A** Dynamics of resources used by individuals of the population. The values on the vertical axis should be multiplied by 10^4^ to obtain the values occurring in the simulations. **B** Population dynamics. The population goes extinct at time step 11

Population dynamics of identical individuals is very simple (Fig. 2). It starts with a low number of individuals and a relatively high resource level. In this situation, each individual can produce more than one juvenile, and the population size increases in successive time steps. However, the resources which are exploited by increasing number of individuals start shrinking. Thus, the production of juveniles is declining. At a certain time step, it equals one, and this is the case of all individuals in the population, as they are identical, and juvenile production can be expressed only as natural numbers and zero. With a further decline in the amount of resources, juvenile production drops to zero, and no individual can produce juveniles. The population goes extinct. This pattern of population dynamics will hold, with only small differences in the time of extinction, for different values of the model parameters.

#### A single Population. Variable Individuals

**Fig. 3:**
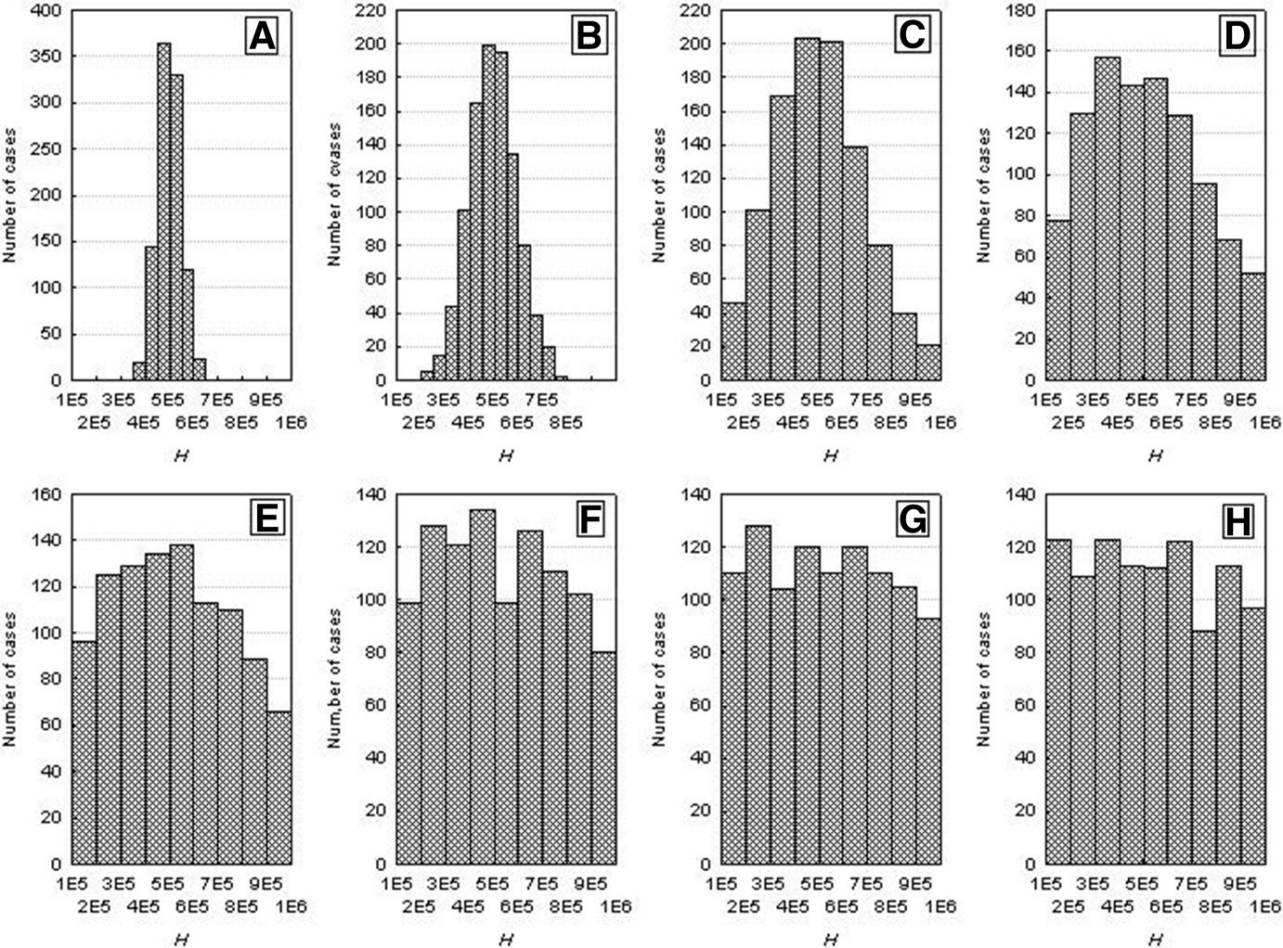
Distributions of the values of *H* parameter for different *H*_*variance*_ values of the truncated normal distribution: **A** *H*_*variance*_ = 50 × 10^3^, **B** *H*_*variance*_ = 100 × 10^3^, **C** *H*_*variance*_ = 200 × 10^3^, **D** *H*_*variance*_ = 300 × 10^3^, **E** *H*_*variance*_ = 400 × 10^3^, **F** *H*_*variance*_ = 500 × 10^3^, **G** *H*_*variance*_ = 600 × 10^3^, **H** *H*_*variance*_ = 700 × 10^3^. The mean of the normal distribution *H*_*mean*_, from which *H* values were drawn equals 500 × 10^3^. The drawn values of *H* cannot be lower than *H*_*min*_ = 100 × 10^3^ and higher than *H*_*max*_ = 1000 × 10^3^

Figure 3 illustrates the frequency distributions of the half saturation constant *H* from Eq. (4). These values were drawn from a double-sided truncated normal distribution. For low values of the variance, the variation of *H* is low, it increases with increasing variance, and for its high values *H* is almost evenly distributed within the acceptable range. The population consists now of variable individuals with lower and higher values of the parameter *H*. For a constant level of resources, an individual with a low value of this parameter will be characterized by a higher assimilation, and consequently, by a greater body weight and juvenile production than an individual with higher values of these variables (see Fig. 1B).

**Fig. 4:**
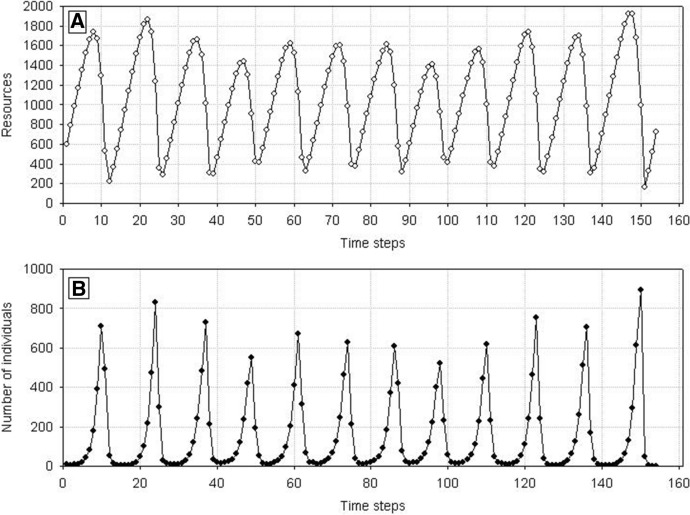
Dynamics of a population composed of variable individuals. Standard parameter values, *N*_0_ = 10 and *H*_*variance*_ = 300 × 10^3^. **A** Dynamics of resources used by individuals in the population. Numbers on the vertical axis should be multiplied by 10^4^. **B** Population dynamics. The population goes extinct at time step 153

Now, in a longer time perspective, the population dynamics will be significantly different from the dynamics of a population made up of identical individuals, although the initial phases will be similar (Fig. 4). After the first maximum, the population size and resource level start declining. However, now the population does not go extinct after reaching the minimum numbers. This is so because in the population comprising variable individuals also at a low resource level there will be at least one individual with a correspondingly low value of the parameter *H* whose weight will be sufficient for the production of at least one juvenile. As the number of individuals is low, then resources are exploited at a low rate and, constantly supplemented, they start increasing. This is followed by an increase in the population size, and the cycle is repeated. In this way, the population can go through several cycles of growth and decline. However, sooner or later, it may happen that in the phase of a low number of individuals and low resource level there will be no individual with a correspondingly low value of the parameter *H* for juvenile production, and the population will go extinct.

**Fig. 5:**
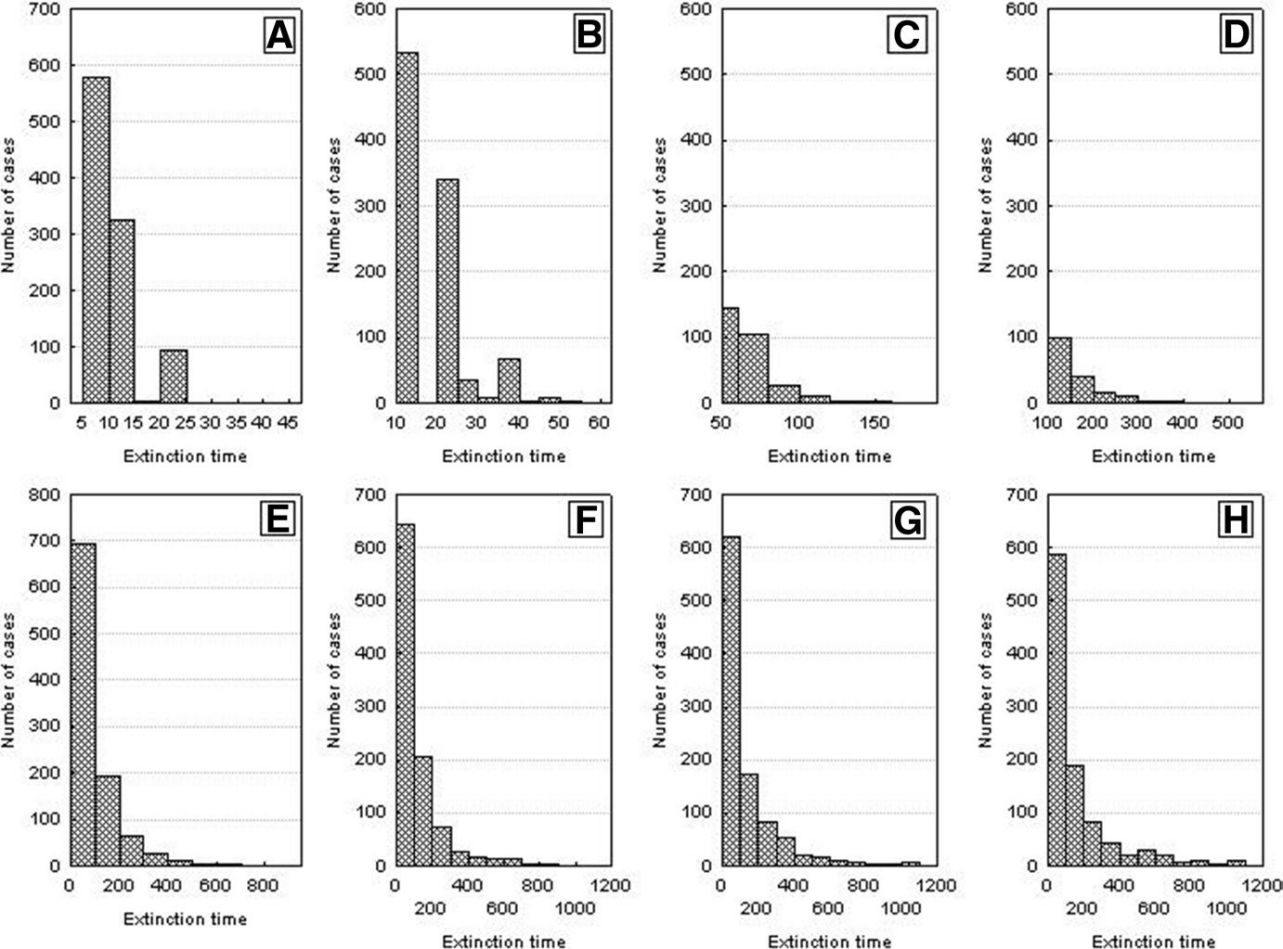
Distributions of the population extinction times for different *H*_*variance*_. Other parameters have standard values. Results of 1000 repeat simulation runs. **A** *H*_*variance*_ = 50 × 10^3^. **B** *H*_*variance*_ = 100 × 10^3^, **C** *H*_*variance*_ = 200 × 10^3^, **D** *H*_*variance*_ = 300 × 10^3^, **E** *H*_*variance*_ = 400 × 10^3^, **F** *H*_*variance*_ = 500 × 10^3^, **G** *H*_*variance*_ = 600 × 10^3^, **H** *H*_*variance*_ = 700 × 10^3^

The time when this occurs will be much longer than for the population comprising identical individuals. Figure 5 shows the frequency distributions of the extinction times of a population for different values of *H*_*variance*_, based on 1000 repeated simulation runs. Increasing individual variation accounts for an increased extinction time. At low individual variation, extinction times are similar to those for populations of identical individuals. When individual variation was increasing, the times to population extinction were increasing and approached several hundred time steps for high values of *H*_*variance*_. However, the distributions of the extinction times were positively skewed all time, with a predominance of the relatively shortest times.

#### Two Competing Species

**Fig. 6:**
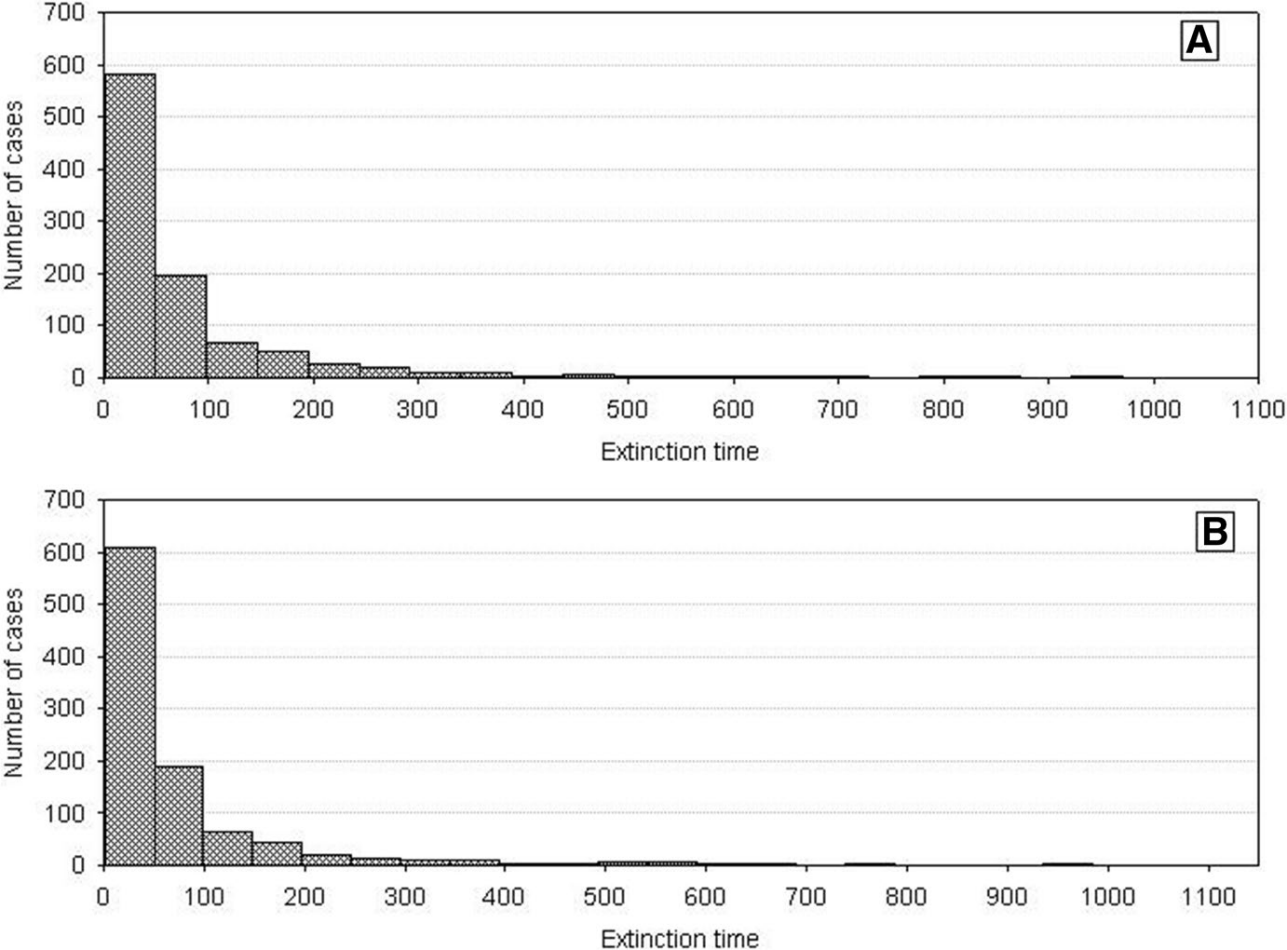
Distributions of the population extinction times for two competing species with equal and large variations *H*_*variance*_^1^ = *H*_*variance*_^2^ = 650 × 10^3^. The other parameters have standard values. The results of 1000 repeat simulation runs. **A** Species 2, **B** species 1

**Fig. 7:**
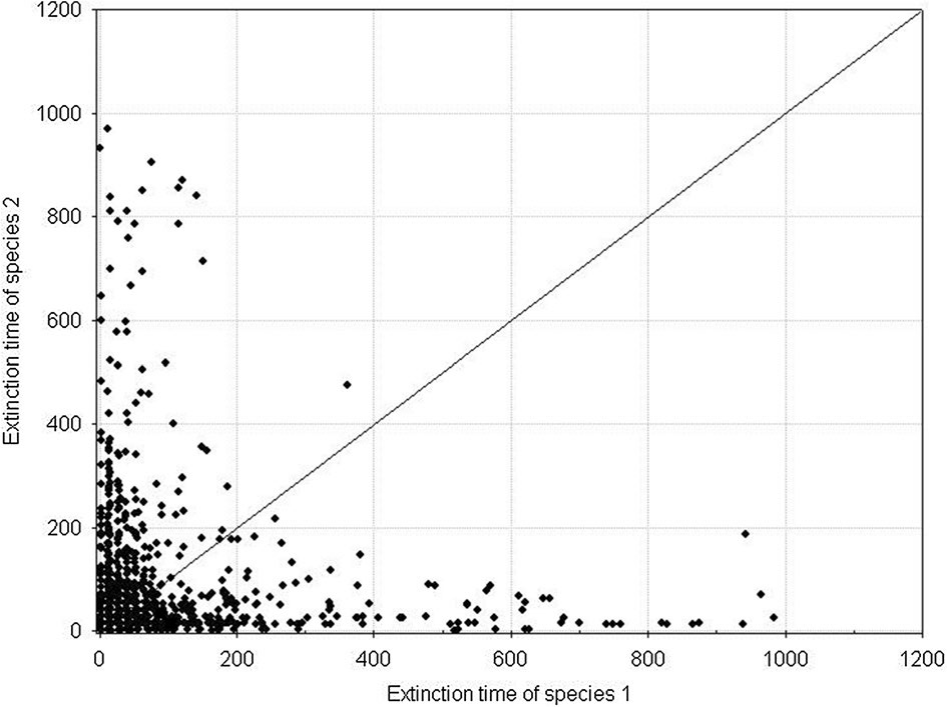
Pairwise comparison of the extinction times for competing species. The species have equal but large variances: *H*_*variance*_^1^ = *H*_*variance*_^2^ = 650 × 10^3^. The other parameters have standard values. The results of 1000 repeat simulation runs

Let us begin with the number dynamics of two competing species characterized by identical but large variances *H*_*variance*_^1^ = *H*_*variance*_^2^. Frequency distributions of the extinction times of each of them are shown in Fig. 6. They are similar. The paired two-sample Wilcoxson test indicates that the mean values of these distributions are not statistically different. Thus, we cannot say that one of them has a longer extinction time. Figure 7 illustrates the pairwise comparison (extinction time of population 1—extinction time of population 2). Situations when both populations go extinct simultaneously are relatively rare. Most often one of these populations goes extinct earlier. It is important, however, that this happens equally often for each of them.

**Fig. 8:**
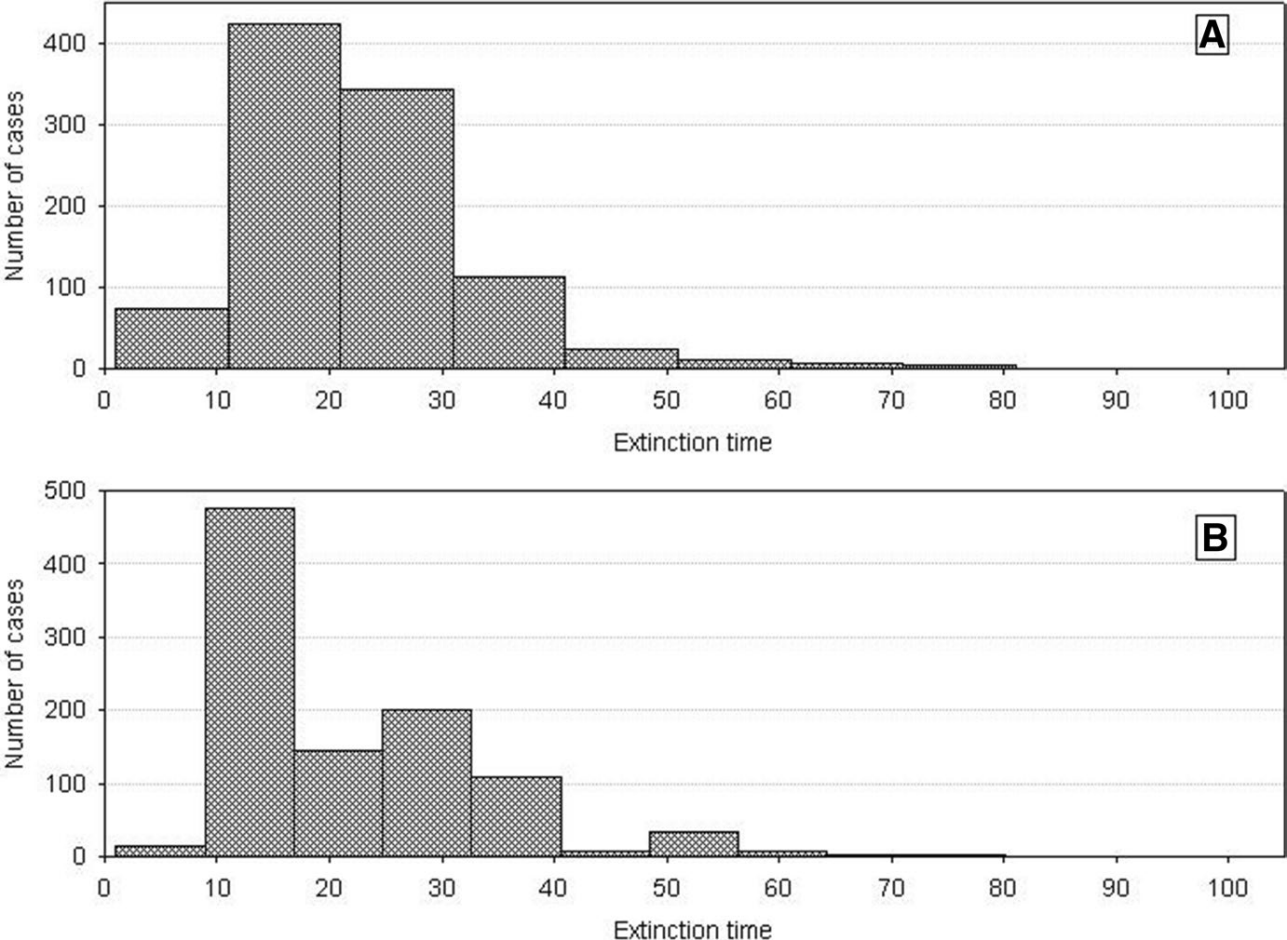
Distributions of the population extinction times for two competing species with equal but small variances *H*_*variance*_^1^ = *H*_*variance*_^2^ = 150 × 10^3^. The other parameters have standard values. The results of 1000 repeat simulation runs. **A** Species 2, **B** species 1

**Fig. 9:**
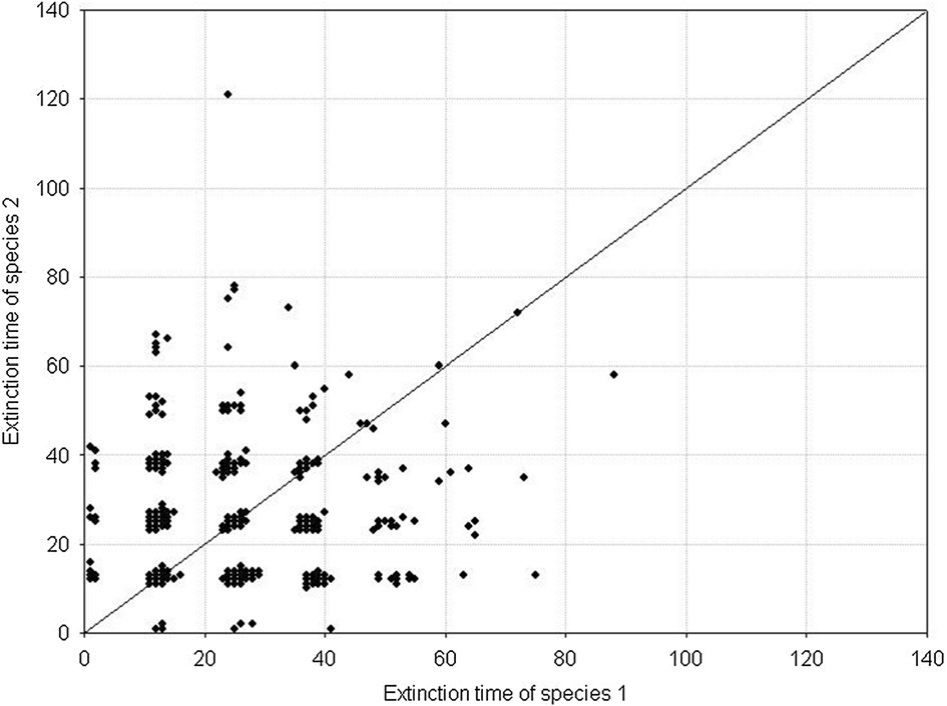
Pairwise comparison of population extinction times for competing species. The species have equal but small variances: *H*_*variance*_^1^ = *H*_*variance*_^2^ = 150 × 10^3^. The other parameters have standard values. The results of 1000 repeat simulation runs

Similar outcomes are when *H*_*variance*_^1^ = *H*_*variance*_^2^ but for low values of the variance. Figure 8 shows the distributions of extinction times of both populations. The Wilcoxson test shows no significant differences in their mean values. Pairwise comparison of extinction times (Fig. 9) is also symmetric about the diagonal. The only difference is that now the extinction times are shorter. This, however, agrees with what we already know about properties of the dynamics of a single population composed of variable individuals.

**Fig. 10:**
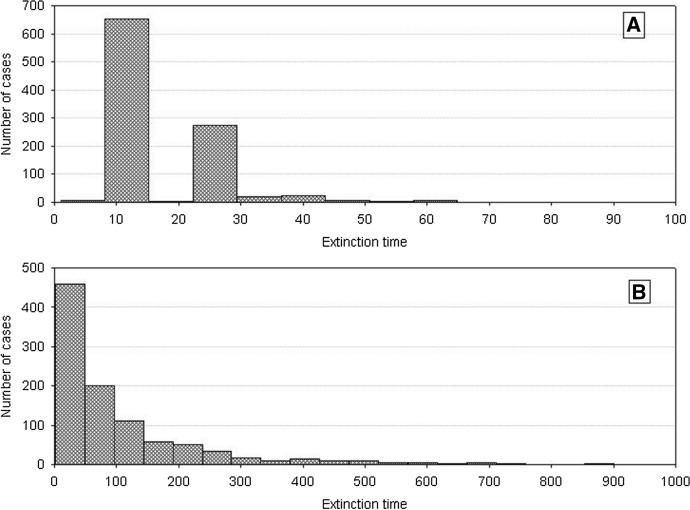
Distributions of population extinction times for two competing species with different and large variances *H*_*variance*_^1^ = 650 × 10^3^ and *H*_*variance*_^2^ = 150 × 10^3^. The other parameters have standard values. The results of 1000 repeat simulation runs. **A** Species 2, **B** species 1

**Fig. 11:**
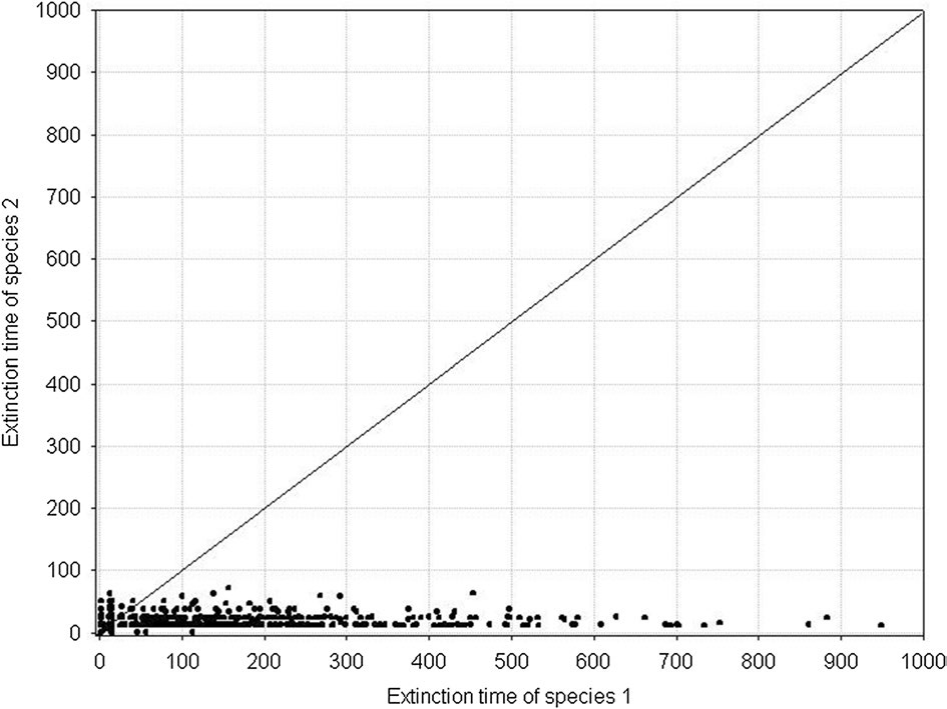
Pairwise comparison of population extinction times for competing species. The species have different but large variances: *H*_*variance*_^1^ = 650 × 10^3^ and *H*_*variance*_^2^ = 150 × 10^3^. The other parameters have standard values. The results of 1000 repeat simulation runs

A different situation emerges when *H*_*variance*_^1^ ≠ *H*_*variance*_^2^. Figure 10 shows the distributions of extinction times in the case of a large difference in the variances between the two species. The more variable population has longer extinction times. The Wilcoxson test shows significant differences in their mean values, and the pairwise comparison of extinction times is clearly asymmetric about the diagonal in favor of the species with greater individual variability (Fig. 11). The extinction times of the latter can be very long compared to those of its competitor.

**Fig. 12:**
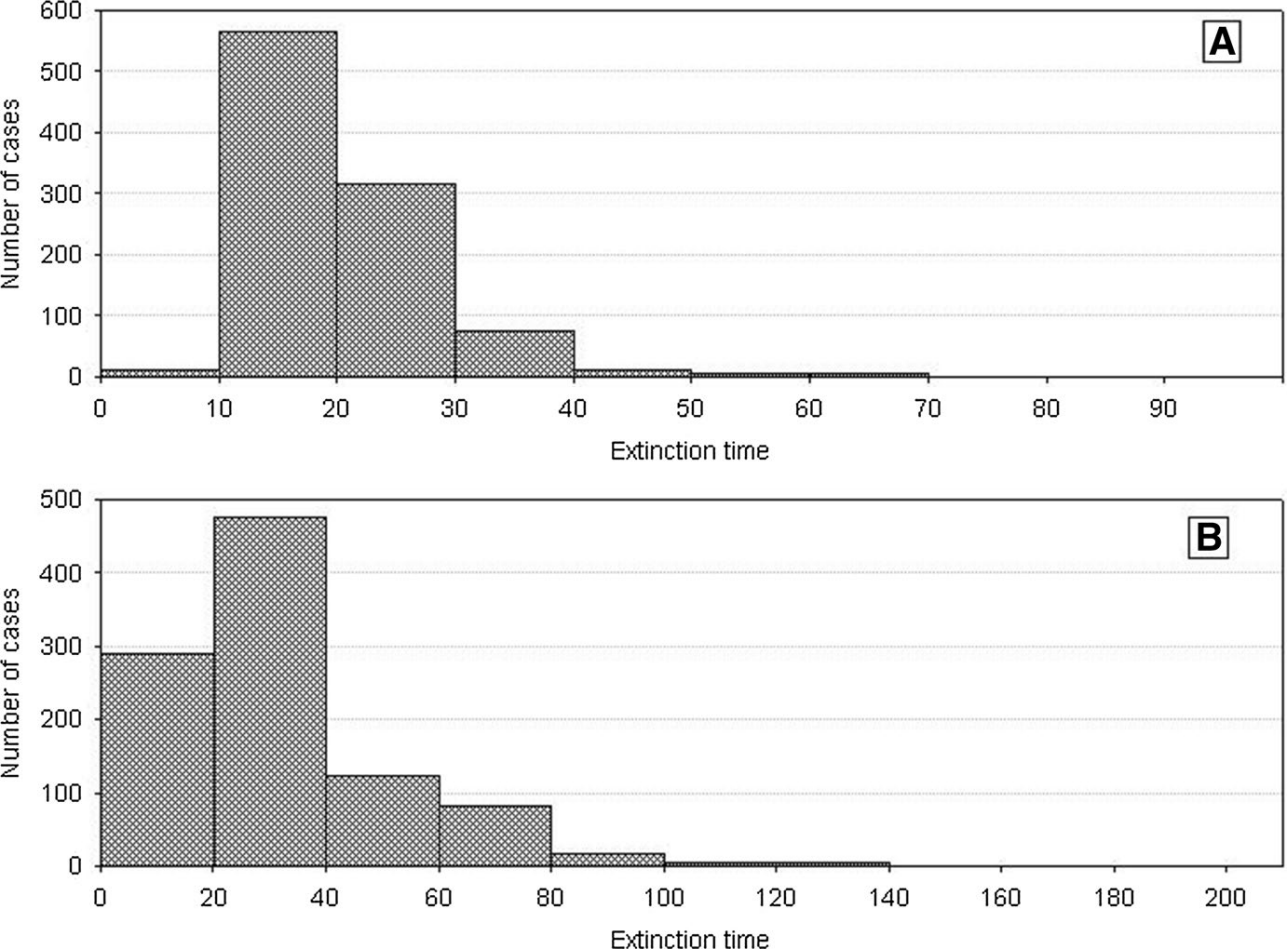
Distributions of extinction times of the populations of two competing species with different and small variances: *H*_*variance*_^1^ = 200 × 10^3^ and *H*_*variance*_^2^ = 150 × 10^3^. The other parameters have standard values. The results of 1000 repeat simulation runs. **A** Species 2, **B** species 1

**Fig. 13:**
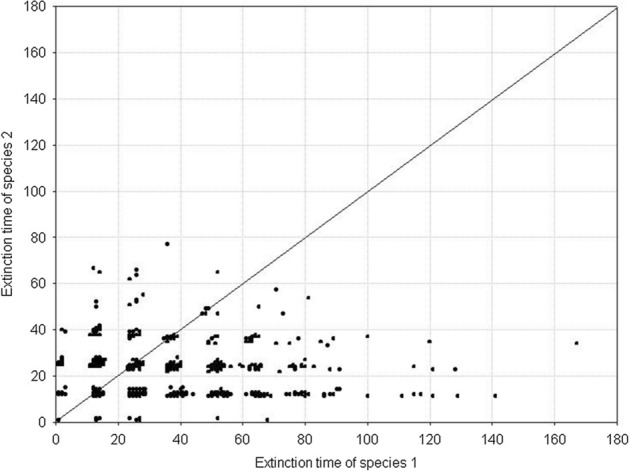
Pairwise comparison of population extinction times for competing species. The species have different but small variances: *H*_*variance*_^1^ = 200 × 10^3^ and *H*_*variance*_^2^ = 150 × 10^3^. The other parameters have standard values. The results of 1000 repeat simulation runs

We obtain the same results when *H*_*variance*_^1^ ≠ *H*_*variance*_^2^ but the difference in variances is smaller than previously. Mean values of the distributions of extinction times are significantly different (Fig. 12), and the pairwise comparison of extinction times is asymmetric about the diagonal as Fig. 13 shows, with a dominance of the extinction times of species whose individuals are more variable. The only difference compared with the preceding case is that the extinction times of both populations are much shorter. Moreover the differences in the extinction times between pairs of competing species are smaller.

**Fig. 14:**
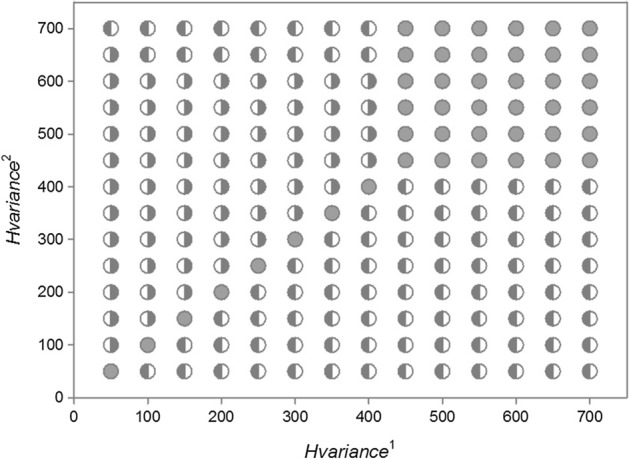
Population dynamics of two competing species presented in the parameter space *H*_*variance*_^1^ and *H*_*variance*_^2^. The other parameters have standard values. The numbers on the two axes should be multiplied by 10^3^ to obtain the real values of the two variances. Gray circles—the two species coexist—the population of species 1 goes extinct first with the same frequency as the population of species 2. Gray left-half of the circle—species 1 is the winner of the competition, the population of species 1 has a longer extinction time more often than the population of species 2. Gray right-half of the circle—species 2 is the winner of the competition, the population of species 2 has a longer extinction time more often than the population of species 1

Figure 14 shows all possible results characterizing the population dynamics of two competing species differing in individual variability. Across the full range of *H*_*variance*_^1^ and *H*_*variance*_^2^ values presented in the graph, if only *H*_*variance*_^1^ = *H*_*variance*_^2^, the competing populations coexisted in the sense presented in Figs. 7 and 9: the distributions of their extinction times did not differ, and after multiple replications of simulation runs, population 1 went extinct the same number of times as did population 2. Figure 5 shows that for a large individual variability, population extinction times become very long (population persistence is very high). For this reason, two competing populations with high individual variation (upper- right corner in Fig. 14) can coexist (in the above meaning of this term) even when they differ in the values of *H*_*variance*_^1^ and *H*_*variance*_^2^, although these differences should not be too large. If they are too large, the population composed of more variable individuals (lower-right and upper-left corners in Fig. 14) will have much more often longer extinction times. However, when individuals of both populations are characterized by small individual variability (lower-left corner in Fig. 14), even a small deviation from the equality of variances *H*_*variance*_^1^ and *H*_*variance*_^2^ causes that the population with more variable individuals wins the competition.

## Individual Variability as an Effect of Intraspecific Competition

### Model of the Dynamics of a Single Population

The algorithm described earlier in Sect. 2 was used here with the same power functions describing the assimilation [Eq. (1)], respiration [Eq. (2)], and the growth equation [Eq. (3)] of an individual. However, different functions were used to describe dependence of food assimilation of an individual on food availability and resource partitioning among competing individuals.

As earlier, the rate of assimilation depends on the amount of food available. The rate of assimilation *A* of a single individual isolated from interactions with other individuals of the same species, as a function of the amount of food *V* can be described by the equation proposed by Ivlev (1961):

12 $$A = a_{{{1},max}} ({1 }{-}e^{ - sV} )w^{b_1}$$

where *a*_1,*max,*_ as earlier, is the maximal value of parameter *a*_1_ reached when *V* = ∞ and *s* is constant parameters describing the rate of reaching this maximal value.

However, if individuals feed together, they may compete for food. We assume that this is a global competition. Each individual competes with all other individuals in the population by using the common food supply. This leads to uneven food partitioning among competitors. If individuals often compete, then the individual who acquired more resources in the past, will acquire more of them also in the future. A good measure of the amount of resources acquired by an individual in the past, accounting also for the energy costs of resource acquirement, is its actual weight. For this reason, the rate of assimilation of an individual in the case of a group of competing individuals is described by Eq. (12) with additionally added dependence on the actual body weight of the individual according to the scheme below.

At each simulation step, individuals with the lowest weight *w*_*min*_ and the highest weight *w*_*max*_ are identified. The rate of assimilation by the lightest individual is described as

13 $$A = a_{{1,\max }} (1 - e^{{ - s_{min} V}} )w_{{\min }}^{{b_1}}$$

and that of the heaviest individual as

14 $$A = a_{{1,\max }} (1 - e^{{ - s_{max} V}} )w_{{\max }}^{{b_1}}$$

**Fig. 15:**
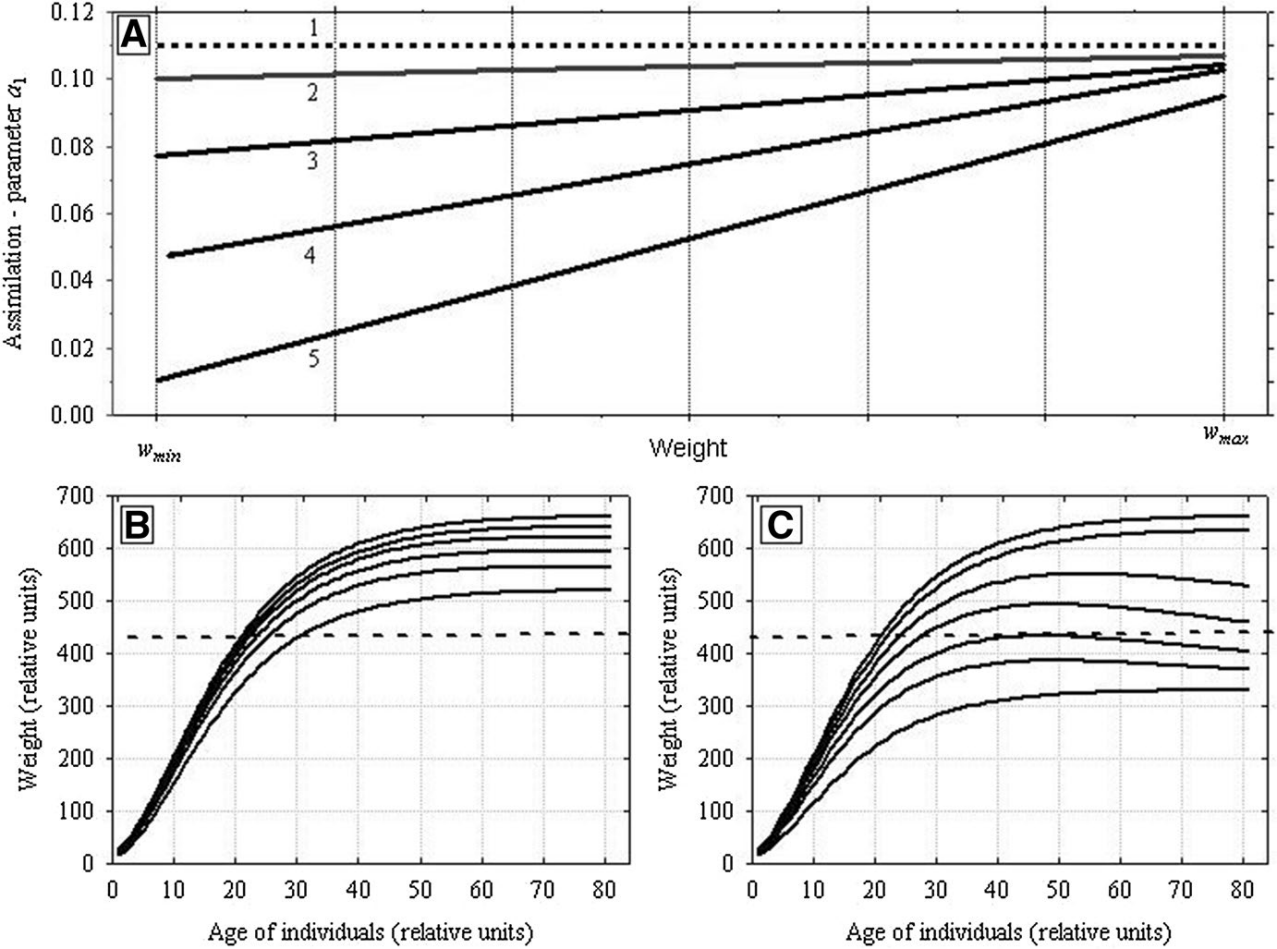
Resource partitioning among competing individuals and their growth in relation to the amount of food. **A** Illustrates how the value of parameter *a*_1_ was calculated for individuals that differed in the actual body weight. *w*_*min*_ and *w*_*max*_ are the lowest and the highest weights of an individual in the current population. Sections of straight lines represent linear approximations applied for calculating the values of parameter *a*_1_ for individuals with body weights greater than *w*_*min*_ and smaller than *w*_*max*_ at different food resources *V*. (1)—food amount *V* = ∞. Values of the parameter *a*_1_ are the same for all individuals in the population and equal to the maximum value *a*_*max*_. Successive lines (2, 3, and 4) illustrate the values of *a*_1_ at declining food amounts *V*. It can be seen that differences among individuals in food consumption are increasing with declining *V.* However, the decline in food supply triggers a considerably greater decline in assimilation by individuals with lower body weights than with higher weights. **B**, **C** Growth curves of individuals obtained by applying the method presented above for calculating the assimilation rates of competitors. The results of growth simulation are shown for several chosen individuals at a constant food supply *V* (**B** *V* = 6 × 10^6^, **C** *V* = 4 × 10^6^). It can be seen that differences in body weights increased with age and they were greater at lower levels of food supply. Horizontal dashed line shows the threshold value of the weight *w*_*fak*_* w*_*max*_^*end*^. Individuals with the final weight higher than the threshold weight will reproduce

The assimilation rates of individuals with intermediate weights are calculated by using linear interpolation between the values from Eq. (13) for *w*_*min*_ and from Eq. (14) for *w*_*max *_(Fig. 15A). As it was stressed in the Sect. 1 the analysis of the weight distributions of growing and at the same time competing individuals shows (Uchmański 1985; Uchmański and Dgebuadze 1990) that to obtain positively skewed distributions, a linear or convex function should be used for the interpolation. The simpler linear case has been chosen in the present model.

Between the values of constant parameters *s*_*min*_ and *s*_*max*_ of Eqs. (13) and (14), there is an inequality

15 $$s_{min} \le s_{max}$$

When *s*_*min*_ = *s*_*max*_, individuals in even-aged population are equal. Assimilation of each individual depends in the same way on *V*. When *s*_*min*_ < *s*_*max*_, individuals differ in the rate of assimilation. The degree of these differences increases with the increasing difference between *s*_*min*_ and *s*_*max*_ or decreasing *V*. However, the differences disappear for *V* → ∞ (Fig. 15A). The heaviest individual at each time step (*w*_*max*_) and at the end of growth (*w*_*max*_^*end*^) was a hypothetic individual growing under conditions *V* = ∞..

Let the number of individuals in the population be *N*_0_ at the initial time step. The initial weights of individuals are derived from the normal distribution with the mean value *w*_0*,mean*_ and variance *w*_0,*variance*_. They values are confined to the interval [*w*_0*,min*_, *w*_0*,max*_] located symmetrically around *w*_0*,mean*_. At each time step, the lowest and the highest weights of individuals in the population are identified. This makes it possible to calculate the assimilation of each individual from the population with respect to its actual weight and to the actual growth conditions. A growth curve can be assigned to each individual. When summed for a generation of even-aged competing individuals, the growth curves form a characteristic “fan” (Fig. 15B and C): differences in body weights between individuals increase with time, and their magnitude depends on growth conditions. The weight distribution at the beginning of the generation is symmetric. However, at the end of the generation it becomes positively skewed. All of this properly simulate the results of many experiments and observations of the growth of competing individuals in even-aged populations (Uchmański 1985).

As earlier in the model with random individual variability, the number of progeny produced by an individual after the end of growth is proportional to the difference between the adult individual weight (*w*_*end*_) and a threshold weight (*w*_*fak*_* w*_*max*_^*end*^) [see Eq. (7)]. The initial juvenile weights of each individual are taken from the normal distribution with a mean *w*_0*, mean*_ and variance *w*_0,variance_, and as earlier their values should be in the range [*w*_0*,min*_,*w*_0*,max*_].

The model describes the dynamics of a population with nonoverlapping generations. Adult individuals die after giving birth, and their progeny form the next generation, the individuals of which grow and reproduce according to the scheme above. The relationship between the numbers of individuals in two subsequent generations is given by Eq. (8).

At the initial time instant, individuals had an amount *V*_0_ of food available. It is assimilated at a rate equal to the sum of the assimilation rates of all individuals, and it regenerats at a constant rate *g* [see Eq. (9)].

Two categories of time steps were used in this model in the same way as they have been applied in the previous model. The basic simulation step was used for the calculation of the number of individuals in the population of successive generations. Within each generation, 80 smaller time steps were used for calculating the body weights of individuals, the amount of food in the environment, and the values of all variables needed to calculate assimilation by individuals, thus, for example, the lowest body weight *w*_*min*_ and the highest body weight *w*_*max*_ of individuals at a given small time step within the generation.

### Two Competing Species

In the model of competition between two species, the above scheme describing the dynamics of a single population is doubled. As the effect of individual variability on the outcome of competition was analyzed, then the values of all parameters of species 1 and 2 were the same, except for those influencing individual variability. Thus, the species differed in the values of parameters *s*_*min*_ and *s*_*max*_. For species 1, this was the set *s*_*min*_^1^ and *s*_*max*_^1^, and for species 2 *s*_*min*_^2^ and *s*_*max*_^2^. Table 2 shows the values of the model parameters used in the simulations.

**Table 2:** Standard values of the model parameters used in the simulation of competition between two species

	Parameter	Value
Growth equation parameters	*a*_1,*max*_	0.11
–	*a*_2_	0.03
–	*b*_1_	0.7
–	*b*_2_	0.9
Parameters of initial weight distribution	*w*_0,*min*_	14
–	*w*_0,*max*_	26
–	*w*_0,*mean*_	20
–	*w*_*0,variance*_	5
Parameters of resource partitioning function	*s*_*min*_	10 × 10^–8^ to 60 × 10^–8^
–	*s*_*max*_	40 × 10^–8^–130 × 10^–8^
Threshold for reproduction	*w*_*fak*_	0.65
Initial number	*N*_0_	5
Initial resources	*V*_0_	6 × 10^6^
Increase of resources	*g*	2 × 10^6^

Indexes of the species are not shown with parameter symbols, as they differ only in the values of *s*_*min*_ and *s*_*max*_. For *s*_*min*_ and *s*_*max*_ the range of their values is shown, as the results of simulations will be presented for different values of this parameter

It is assumed that there are no direct interactions that occur between individuals of different species. Intraspecific competition will lead to the differentiation of individuals within each species according to the scheme presented above. Individuals of different species “feel”, however, their presence because they use the common food supply. Thus, the presence of a competitor influences only the food conditions under which individuals grow. The Eq. (11) describing the dynamics of resources was used in this model.

### Results

#### Dynamics of a Single Population

A single population comprising nonvariable individuals (*s*_*min*_ = *s*_*max)*_) has a characteristic and simple dynamics already analyzed many times (Uchmański 1999, 2000a, b; Grimm and Uchmański 2002) and very similar to the dynamics of nonvariable individuals from Sect. 2. In short, after an initially exponential increase and reaching maximum, the population goes extinct. This pattern of population dynamics does not depend on the initial number of individuals, the initial food supply in the environment, nor on the values of the other parameters of the model. It is only important that the individuals be identical, grow identical, attain the same weight at the end of growth, and, as a result, produce the same number of juveniles. Population dynamics for different values of the model’s parameters will differ only in the time of extinction, but typically it will be short, of the order of several or several dozen generations. Repeated simulations with the same values of parameters will lead to the dynamics with the same time of extinction.

**Fig. 16:**
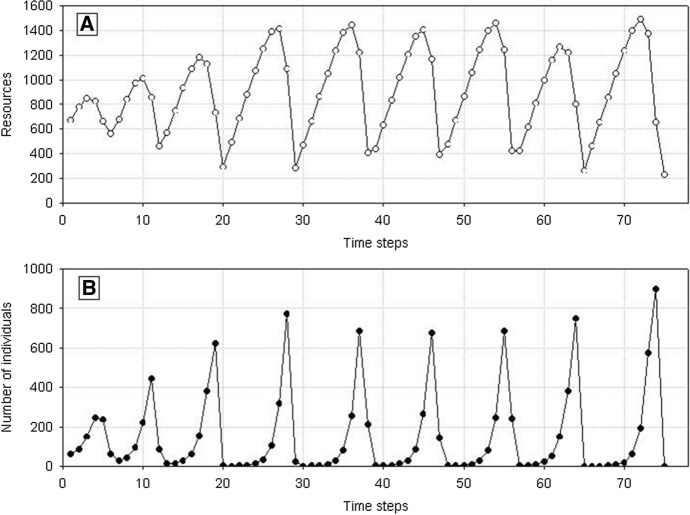
Dynamics of a single population (**B**) and resources (**A**). Individuals differ from each other. Standard values of parameters are used: *s*_*min*_ = 36 × 10^–8^, *s*_*max*_ = 100 × 10^–8^. The population goes extinct at time step 75. Numbers on the vertical line of **A** should be multiplied by 10^4^ to get the values occurring in simulations

A single population comprising variable individuals (*s*_*min*_ < *s*_*max*_), like previously, has much more complex dynamics. After the first maximum, the number of individuals in the population will be decreasing with declining food supply in the environment. However, it will not go extinct after the first minimum, as when the variability of individuals is large enough even at a low level of food, there will be a chance that a sufficiently good individual will be present in the population to produce progeny under such conditions. And when the number of individuals is low, food will be exploited at a low rate, so it will start growing. This pattern will be followed by the population growth up to the next maximum, followed by the next minimum. The population may go through many such cycles, but sooner or later, no individual capable of reproduction will be present at the minimum value. Then also the population of variable individuals will die (Fig. 16). However, the time of population extinction will be much longer than in the case of nonvariable individuals.

**Fig. 17:**
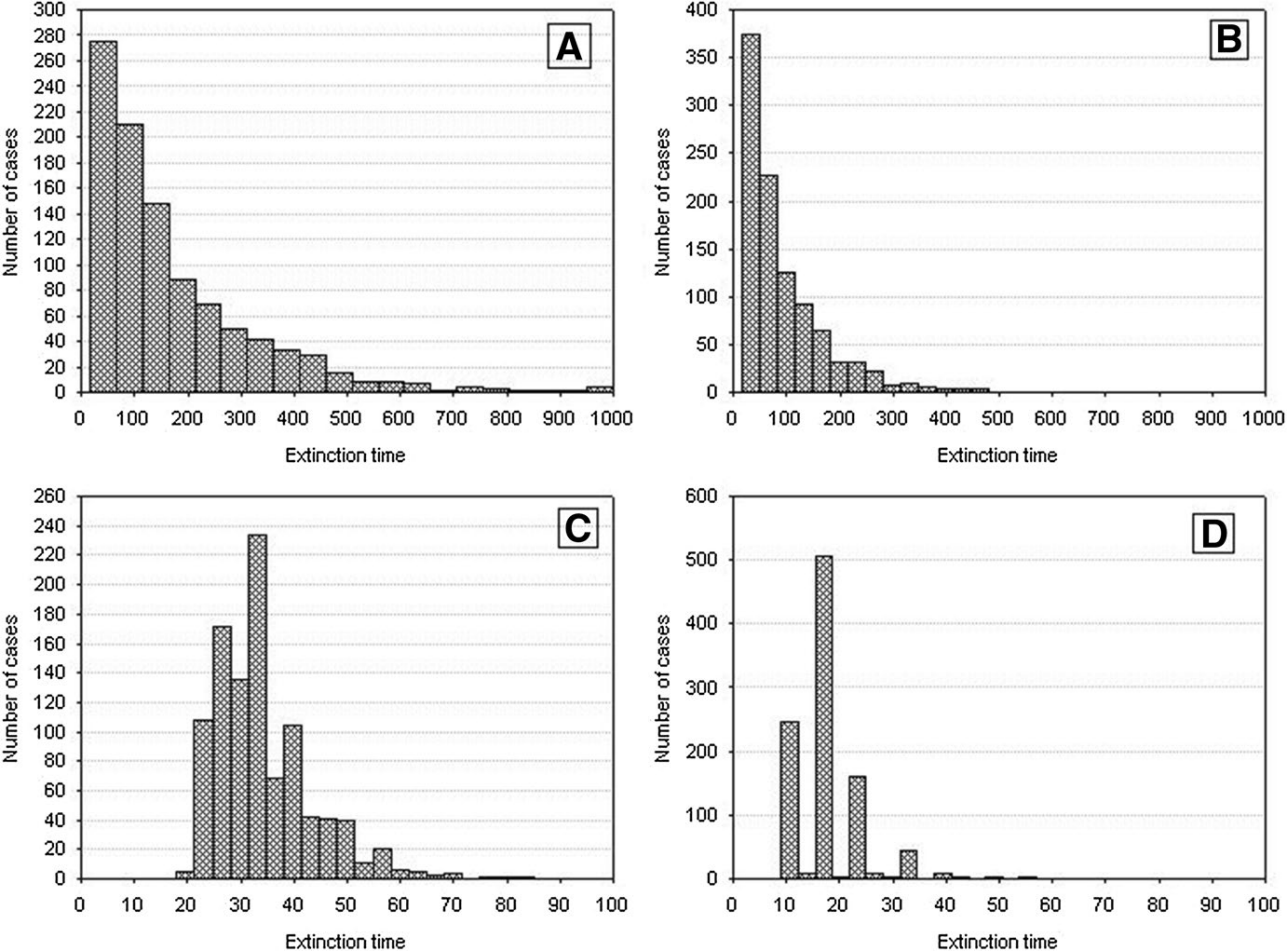
Frequency distributions of extinction times of a single population composed of variable individuals. Results of 1000 runs of simulations with standard values of the parameters. *s*_*max*_ = 100 × 10^–8^. **A** *s*_*min*_ = 33 × 10^–8^, **B** *s*_*min*_ = 36 × 10^–8^, **C** *s*_*min*_ = 40 × 10^–8^, **D** *s*_*min*_ = 50 × 10^–8^. The proportion of long extinction times of the population declines with increasing value of *s*_*min*_

As population extinction at the minimum number is a random event, then repeated simulation runs with the same parameter values will yield dynamics with different extinction times. The distributions of extinction times for various degrees of individual variability (measured as the difference between *s*_*min*_ and *s*_*max*_) are shown in Fig. 17. The proportion of long extinction times clearly increases with increasing individual variability. And it does not matter in which way the increase in the difference *s*_*min*_ and *s*_*max*_ is realized: whether *s*_*min*_ is constant and *s*_*max*_ increases, or vice versa, or in any other way.

#### Two Competing Species: ***s***_***min***_^1^ ≠ ***s***_***min***_^2^

**Fig. 18:**
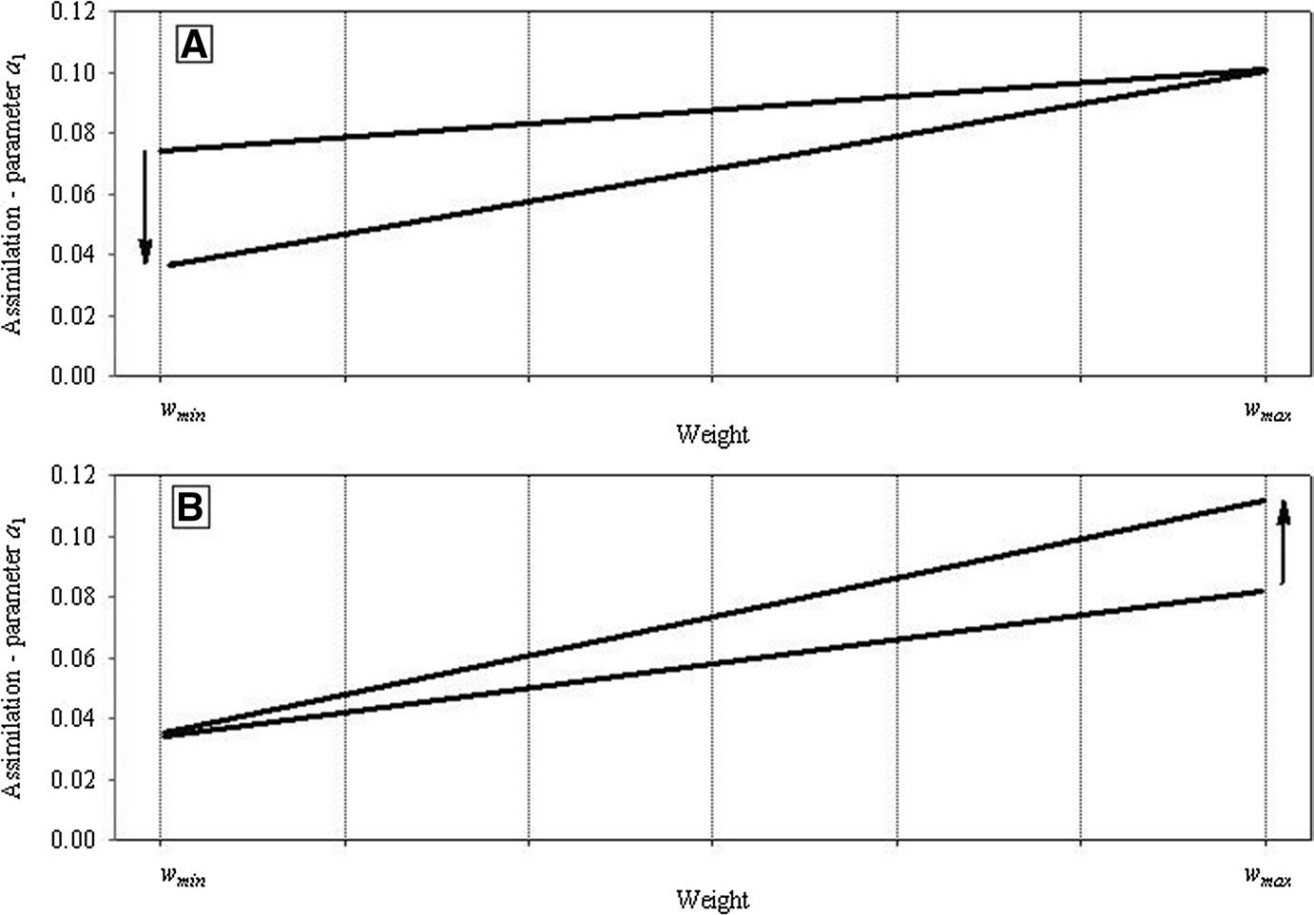
Two competing species. Illustration of the differences in food partitioning among individuals of each population, leading to individual variability in growth rate, weight attained, and number of juveniles produced. This is a replica of the lines from Fig. 15A for each species separately. **A** The first way of individual variability *s*_*max*_^1^ = *s*_*max*_^2^ and *s*_*min*_^1^ ≠ *s*_*min*_^2^. The species with a lower value of *s*_*min*_ is more variable. **B **The second way of individual variability: *s*_*min*_^1^ = *s*_*min*_^2^ and *s*_*max*_^1^ ≠ *s*_*max*_^2^. The species with a higher value of *s*_*max*_ is more variable. Arrows indicate the ways in which greater individual variability in resource assimilation by individuals of a species were incorporated into the model

In the model presented here, two competing species may differ in individual variability in several ways. In this paper, two of them are chosen. In the first case, both species have the same value of the parameter *s*_*max*_ (*s*_*max*_^1^ = *s*_*max*_^2^), and they differ in the parameters *s*_*min*_ (*s*_*min*_^1^ ≠ *s*_*min*_^2^) (Fig. 18A). In the second case, the opposite is true: *s*_*min*_^1^ = *s*_*min*_^2^ and *s*_*max*_^1^ ≠ *s*_*max*_^2^ (Fig. 18B).

**Fig. 19:**
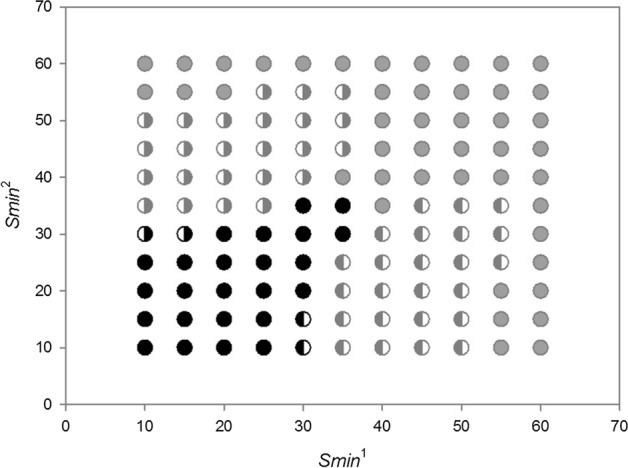
Properties of the dynamics of the system of two competing species in the parameter space *s*_*min*_^1^ and *s*_*min*_^2^. The species have the same values *s*_*max*_^1^ = *s*_*max*_^2^ = 100 × 10^–8^ but they differ in *s*_*min*_^1^ and *s*_*min*_^2^. The other parameters have standard values. Numbers on the axes should be multiplied by 10^–8^ to obtain the parameter values used in simulations. Full-black circles—populations of both species coexist for more than 1000 time steps. Full-gray circles—the two species go extinct simultaneously, but the extinction times are shorter than 1000 time steps. Left half-black circles—species 1 persists longer than 1000 time steps, species 2 goes extinct earlier. Left half-gray circles—species 2 goes extinct earlier, species 1 persists longer, but its extinction time is shorter than 1000 time steps. Right half-black circles—species 2 persists longer than 1000 time steps, species 1 goes extinct earlier. Right half-gray circles—species 1 goes extinct earlier, species 2 persists longer, but its extinction time is shorter than 1000 time steps

**Fig. 20:**
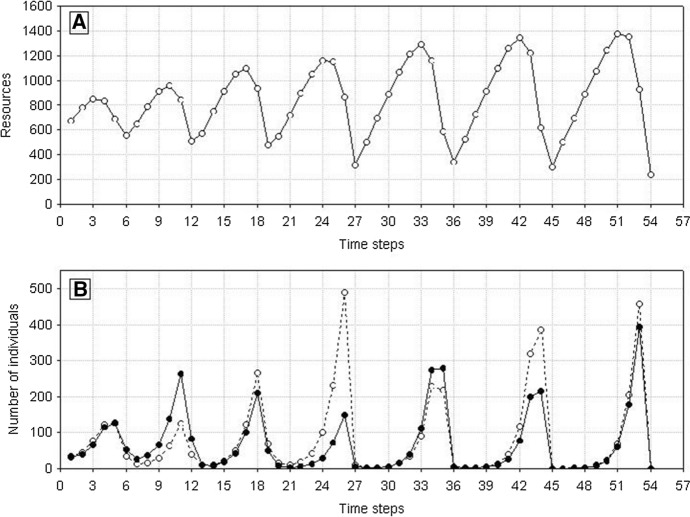
Dynamics of two competing species (**B**) and resources (**A**). Standard values of parameters (Table 2). *s*_*max*_^1^ = *s*_*max*_^2^ = 100 × 10^–8^. Both species have the same individual variability: *s*_*min*_^1^ = *s*_*min*_^2^ = 36 × 10^–8^. Note the synchronization of the dynamics of both populations and similar numbers of individuals. Numbers on the vertical line in **A** should be multiplied by 10^4^ to obtain the values occurring in simulations

**Fig. 21:**
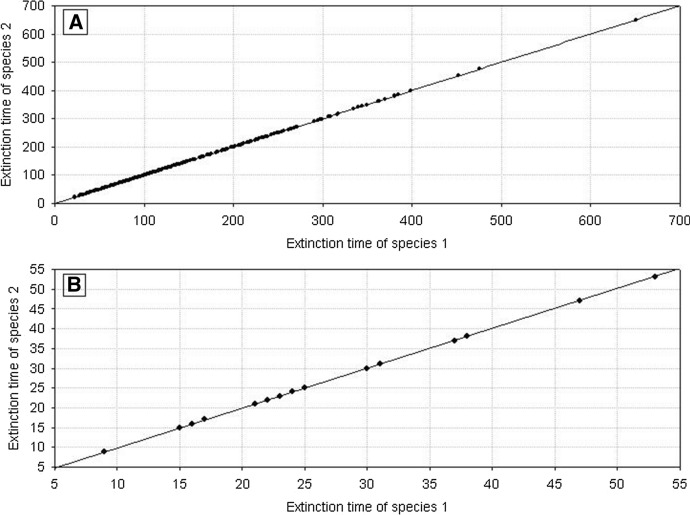
Pairwise comparison of extinction times of species 1 and species 2. Both species are characterized by the same individual variability. **A** *s*_*min*_^1^ = *s*_*min*_^2^ = 36 × 10^–8^, **B** *s*_*min*_^1^ = *s*_*min*_^2^ = 50 × 10^–8^. *s*_*max*_^1^ = *s*_*max*_^2^ = 100 × 10^–8^. The other parameters have standard values (Table 2). In both figures, all points lie on the diagonal, which means that populations go extinct simultaneously. However, the populations with larger individual variability have longer extinction times

Let consider the first case. Summary of different types of dynamics of two competing species in the parameter space *s*_*min*_^1^ and *s*_*min*_^2^ illustrates Fig. 19 If both species are characterized by the same degree of individual variability *s*_*min*_^1^ = *s*_*min*_^2^, they will coexist and die simultaneously. For small but the same values of *s*_*min*_^1^ and *s*_*min*_^2^, what in the case of both these species gives large individual variability, the times of extinction are long, exceeding the maximum value set for simulation, that is, 1000 time steps. For larger but also the same values of *s*_*min*_^1^ and *s*_*min*_^2^, the extinction times are shorter but as in the previous case identical for both species (Figs. 20 and 21).

However, not only the species with the same individual variability may coexist. Also the species that differ in variability have this ability, but under condition that the differences in individual variability are not too large In the lower left part of Fig. 19 there is a quite large area where both species coexist, and the extinction time of each of them exceeds 1000 time steps. In this area, *s*_*min*_^1^ and *s*_*min*_^2^ have low values, which means that both have high individual variability. And this, as we remember from the analysis of the properties of a single population, coincides with a high persistence of each of them. The area of the parameter space where this occurs is in fact the only one where we have to do with a real long-term coexistence of both species.

**Fig. 22:**
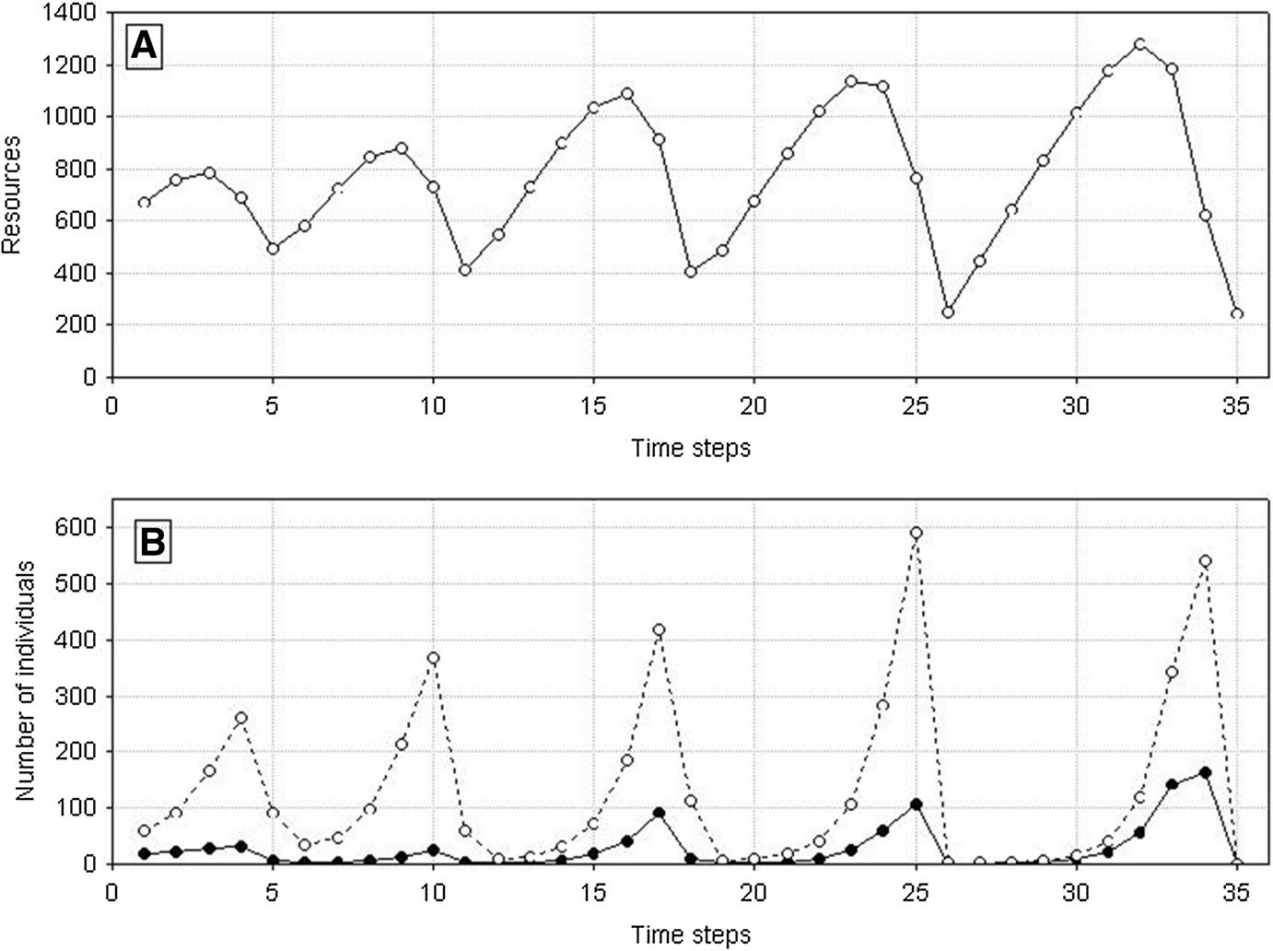
Dynamics of two competing species (**B**) and resources (**A**). Standard values of the parameters (Table 2). *s*_*max*_^1^ = *s*_*max*_^2^ = 100 × 10^–8^. The species differ in their individual variability. Filled circles in **B** illustrate the numbers of species 1 with a higher individual variability *s*_*min*_^1^ = 36 × 10^–8^, Empty circles denote numbers of species 2 with a lower individual variability *s*_*min*_^2^ = 40 × 10^–8^. Nevertheless, these populations go extinct simultaneously. Let us note that the less variable species attain much higher numbers at the maximum. Numbers on the vertical axis in **A** should be multiplied by 10^4^ to obtain the values occurring in simulations

**Fig. 23:**
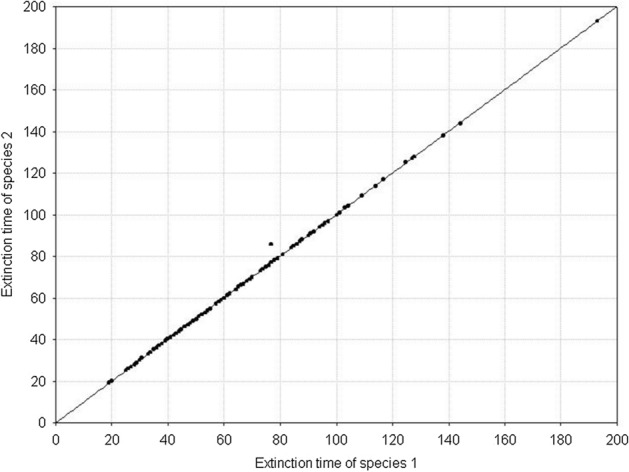
Pairwise comparison of the extinction times of species 1 and 2. The species differ in individual variability: *s*_*min*_^1^ = 36 × 10^–8^, *s*_*min*_^2^ = 40⋅10^–8^. *s*_*max*_^1^ = *s*_*max*_^2^ = 100 × 10^–8^. The other parameters have standard values (Table 2). In spite of differences in variability, all points (except one) lie on the diagonal, which means that the populations of both species go extinct simultaneously

Another such area is in the upper right corner in Fig. 19. However, such populations have short extinction times now. If the differences in individual variability between these two species are not too large, then under conditions of a low food level, they are in a similar situation relatively fast and in the same time step in both populations there are no individuals capable of producing juveniles. Both go extinct at the same, typically short time (Fig. 22 and 23).

Populations of both species coexist and go extinct simultaneously, but again in a short or very short time when the variability of one species is large, and that of the second species is low [lower right corner and upper left corner (Fig. 19)]. Large variability of the species, which is generated by reducing the value of *s*_*min*_, means that differences in the amount of food assimilated by individuals will increase, but this increase is due to an increase in the proportion of individuals with low assimilation and low increases in body weight. Therefore, the proportion of individuals that die before producing offspring increases, and consequently, the population comprising more variable individuals has a low number of individuals. Thus, at the minimum level of population size that they both attain simultaneously, we have similar individuals in one of them, as it is little variable, and in the other population, potentially more variable individuals that can reproduce have no chance to appear since their proportion is low. Consequently, both populations go extinct at the same short time.

**Fig. 24:**
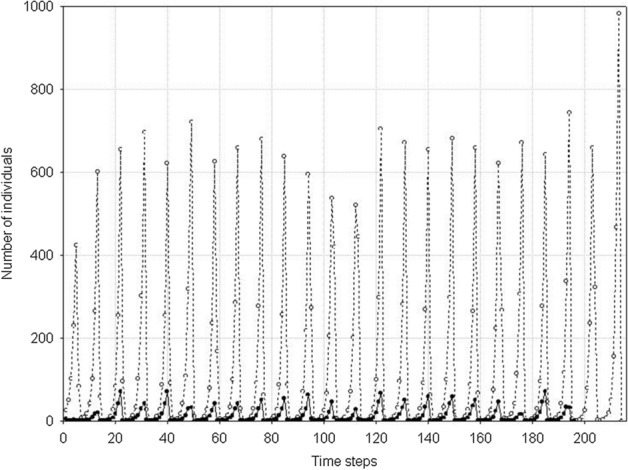
Dynamics of two competing species. Standard values of the parameters (Table 2). *s*_*max*_^1^ = *s*_*max*_^2^ = 100 × 10^–8^. The species differ in their individual variability. Empty circles illustrate the numbers of individuals of species 1 with lower individual variability *s*_*min*_^1^ = 36 × 10^–8^, filled circles illustrate the numbers of species 2 with larger variability *s*_*min*_^2^ = 25 × 10^–8^. The more variable population goes extinct earlier (at time step197), while the population of the less variable species—later (at time step 214). Population of the less variable species attains much higher maxima

**Fig. 25:**
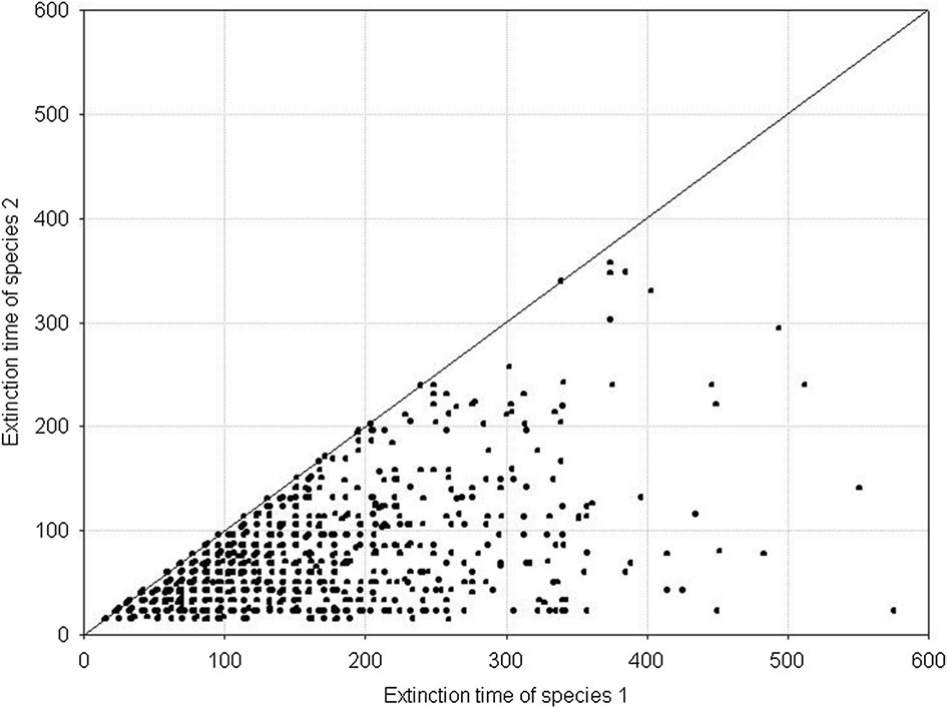
Pairwise comparison of the extinction times of species 1 and species 2. The species differ in individual variability: *s*_*min*_^1^ = 36 × 10^–8^, *s*_*min*_^2^ = 25 × 10^–8^. *s*_*max*_^1^ = *s*_*max*_^2^ = 100 × 10^–8^. The other parameters have standard values (Table 2). Almost all points lie below the diagonal, which means that the population of species 1, with a lower individual variation, has a longer extinction time

However, if the variability of one population remains at the same high level, and the variability of the second population, which was little variable earlier, slightly increases, then a new pattern of the dynamics of the two-species system emerges. In the parameter space shown in Fig. 19, at the central parts of both axes there are areas where, contrary to our intuition, more variable species will be excluded (Figs. 24 and 25). Let us compare these areas of the parameter space with those analyzed in the preceding paragraph. The more variable species continues to be in the same situation. At low numbers, its chance for the realization of potential possibilities resulting from high variability is small. However, the species, which was little variable earlier, now enjoys a higher individual variability at still sufficiently high numbers to take advantage of this chance. That is why the population of the more variable species becomes extinct, and the population of the less variable species survives for a longer time.

If leaving this area, we would continue to increase the variability of the less variable species, then through a narrow boundary area in which the population of the more variable species continues to go extinct earlier, and the time of extinction of the less variable species exceeds 1000 time steps, we would enter the earlier described area of permanent coexistence of both these species, where their extinction times exceed 1000 time steps.

#### Two Competing Species: ***s***_***max***_^1^ ≠ ***s***_***max***_^2^

**Fig. 26:**
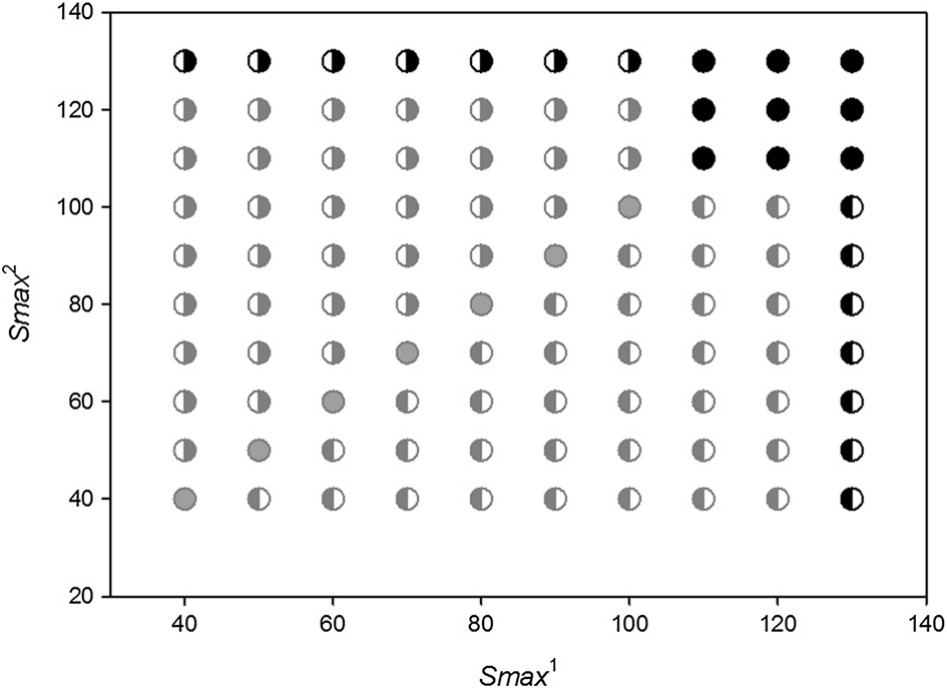
Dynamics of the system comprising two competing species, shown in the parameter space *s*_*max*_^1^ and *s*_*max*_^2^. The species have the same values *s*_*min*_^1^ = *s*_*min*_^2^ = 36 × 10^–8^, but they differ in the values *s*_*max*_^1^ and *s*_*max*_^2^. The other parameters have standard values. Numbers on the axes should be multiplied by 10^–8^ to obtain the parameter values used in the simulations. Full-black circles—populations of both species coexist for more than 1000 time steps. Full-gray circles—the two species go extinct simultaneously, but the extinction times are shorter than 1000 time steps. Left half-black circles—species 1 persists longer than 1000 time steps, species 2 goes extinct earlier. Left half-gray circles—species 2 goes extinct earlier, species 1 persists longer, but its extinction time is shorter than 1000 time steps. Right half-black circles—species 2 persists longer than 1000 time steps, species 1 goes extinct earlier. Right half-gray circles—species 1 goes extinct earlier, species 2 persists longer, but its extinction time is shorter than 1000 time steps

Let’s consider now the second case of individual variability when *s*_*min*_^1^ = *s*_*min*_^2^ and *s*_*max*_^1^ ≠ *s*_*max*_^2^ (Fig. 18B). An overall set of different types of population dynamics of two competing species in the parameter space *s*_*max*_^1^ and *s*_*max*_^2^ is illustrated in Fig. 26. If two species have the same individual variability, *s*_*max*_^1^ = *s*_*max*_^2^, then both these populations become extinct simultaneously. For small values of *s*_*max*_^1^ and *s*_*max*_^2^, when the individual variability of the two species is low, although the same, the extinction times are short. With increasing values of *s*_*max*_^1^ and *s*_*max*_^2^, the extinction times of the two species will be increasing, and for large values of *s*_*max*_^1^ and *s*_*max*_^2^, both populations will exist longer than the maximum simulation time, that is, 1000 time steps. In the upper-right corner of Fig. 26, there is an area indicating that the two populations can exist longer than 1000 time steps not only when *s*_*max*_^1^ = *s*_*max*_^2^ but also when the values of these parameters differ, although slightly. For other ranges of the values of *s*_*max*_^1^ and *s*_*max*_^2^, such areas do not exist. The two populations can go extinct simultaneously only on the condition that *s*_*max*_^1^ = *s*_*max*_^2^.

At all other points of the parameter space where *s*_*max*_^1^ ≠ *s*_*max*_^2^ most results of repeated simulations show that one species goes extinct earlier than the other species and this is the less variable species which goes extinct earlier. At points located below the diagonal in Fig. 26 this will be species 2, while above the diagonal—species 1. At the points located along the right edge of Fig. 12 and along its upper edge, variability of the species, winning the competition is so large that this species exists for more than 1000 time steps, while its competitor dies much earlier.

**Fig. 27:**
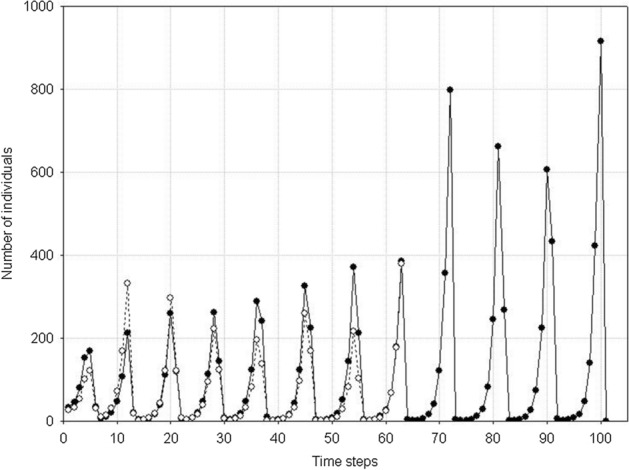
Population dynamics of two competing species. Standard values of the parameters. (Table 2). *s*_*min*_^1^ = *s*_*min*_^2^ = 36 × 10^–8^. The species differ in individual variability. Filled circles denote the number of individuals of species 1, with higher individual variability: *s*_*max*_^1^ = 100 × 10^–8^. Empty circles denote the number of individuals of species 2, which is less variable: *s*_*max*_^2^ = 70 × 10^–8^. The less variable population goes extinct earlier (at time step 64) than the population of the more variable species (at time step 101). As long as they coexist, the numbers of individuals of the two species are similar. After the extinction of the competitor, the population of the more variable species has access to a greater amount food, and it increases

**Fig. 28:**
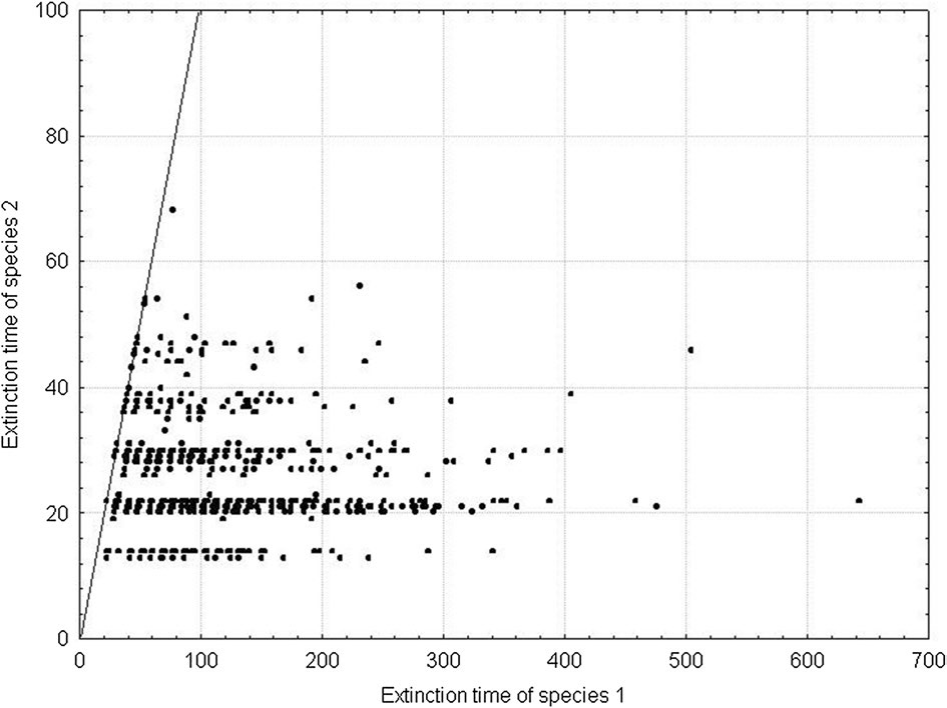
Pairwise comparison of extinction times of species 1 and species 2. The species differ in individual variability: *s*_*max*_^1^ = 100 × 10^–8^, *s*_*max*_^2^ = 70 × 10^–8^. *s*_*min*_^1^ = *s*_*min*_^2^ = 36 × 10^–8^. The other parameters have standard values (Table 2). Most points lie below the diagonal, which means that the population of species 1, with a higher individual variability, has a longer extinction time

Figure 27 illustrates an example of the dynamics of two populations for one pair of the values of parameters *s*_*max*_^1^ and *s*_*max*_^2^ located below the diagonal in Fig. 26. Summary of the extinction times for pairs of competing species with the same parameter values as in Fig. 27 and 1000 repeated simulations is shown in Fig. 28. Some points lie on the diagonal (they are few compared with 1000 simulations) indicating that the populations go extinct simultaneously. However, the majority of points lie below the diagonal, where the more variable species 1 wins the competition.

**Fig. 29:**
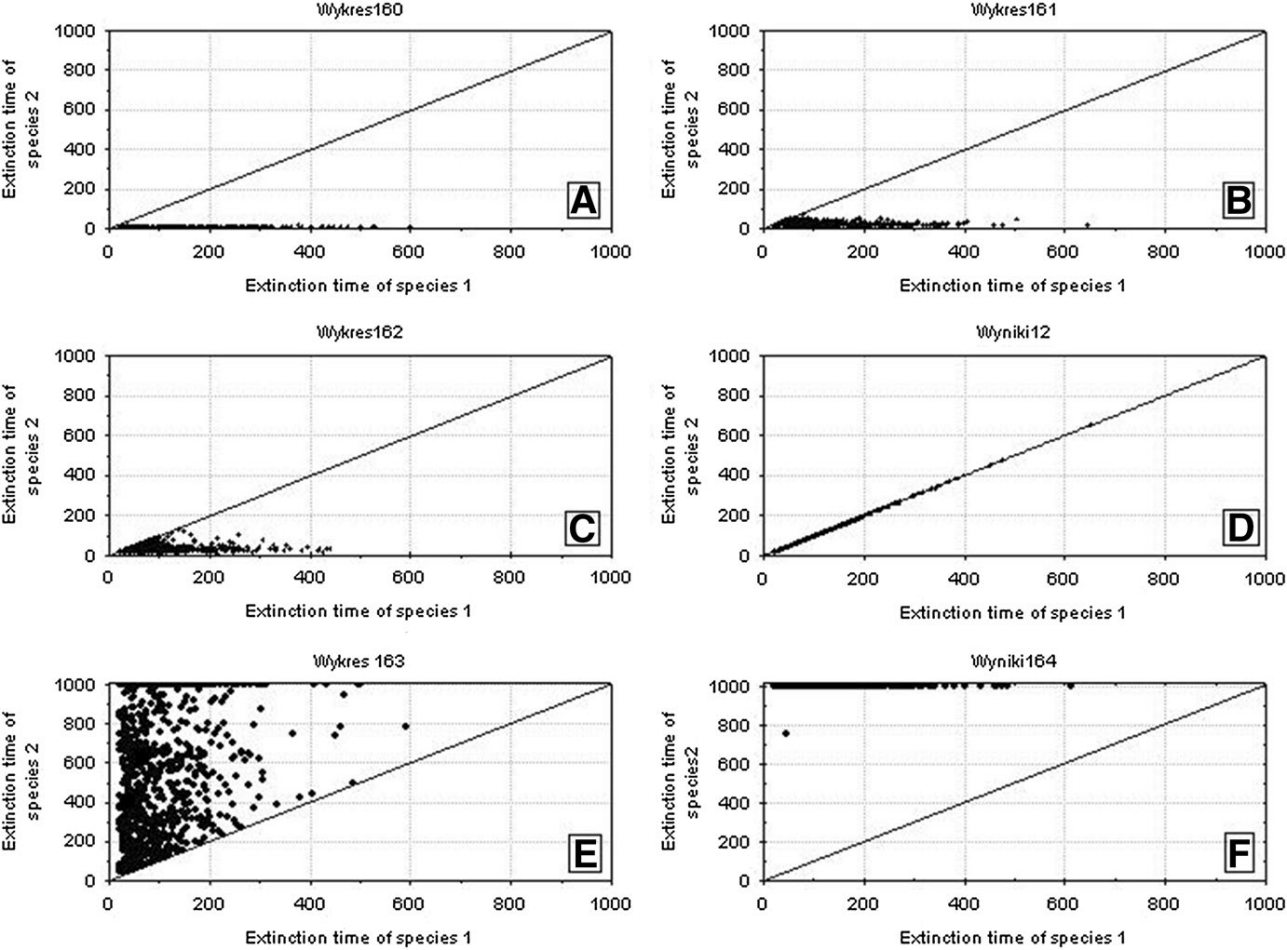
Pairwise comparison of extinction times of species 1 and 2 inrelation to differences between *s*_*max*_^1^ and *s*_*max*_^2^. **A** *s*_*max*_^1^ = 100 × 10^–8^ and *s*_*max*_^2^ = 50 × 10^–8^, **B** *s*_*max*_^1^ = 100 × 10^–8^ and *s*_*max*_^2^ = 70 × 10^–8^, **C** *s*_*max*_^1^ = 100 × 10^–8^ and *s*_*max*_^2^ = 90 × 10^–8^, **D** *s*_*max*_^1^ = 100 × 10^–8^ and *s*_*max*_^2^ = 100 × 10^–8^, **E** *s*_*max*_^1^ = 100 × 10^–8^ and *s*_*max*_^2^ = 120 × 10^–8^, **F** *s*_*max*_^1^ = 100 × 10^–8^ and *s*_*max*_^2^ = 130 × 10^–8^. The other parameters have standard values (Table 2) *s*_*min*_^1^ = *s*_*min*_^2^ = 36 × 10^–8^

Figure 29 shows the location of points with coordinates corresponding to the extinction times of competing species in relation to the differences between *s*_*max*_^1^ and *s*_*max*_^2^. If the values of *s*_*max*_^1^ and *s*_*max*_^2^ differ, then the species with larger individual variability, that is, with a higher value of *s*_*max*_, will survive for a longer time (Fig. 29A). If the differences between *s*_*max*_^1^ and *s*_*max*_^2^ are smaller, then most often the more variable species wins the competition, but in some simulations both these species go extinct simultaneously (Fig. 29B, C and E). However, the contribution of such events is not large, and it declines with increasing difference between *s*_*max*_^1^ and *s*_*max*_^2^. In the extreme case, when the difference between *s*_*max*_^1^ and *s*_*max*_^2^ is large, and when one of the species is characterized by a large value of this parameter, and the second one is not much higher than the corresponding value of the parameter *s*_*min*_, then the less variable species becomes extinct almost immediately, and the more variable species persists more than 1000 time steps (Fig. 29F). If the values of these parameters are equal, then the species coexist and go extinct simultaneously (Fig. 29D).

## Conclusions

A more variable species wins the competition. This is the main result of earlier papers which I reviewed in the introduction. The individual-based models which I described in this paper provide a strong support for this statement, but also a much more precise explanation of it. In classical models, we are forced to analyze the stability properties of the system and the asymptotic behavior of the model solutions. In individual-based models, the situation is different. Now, we are forced to analyze the phenomenon of extinction: the population extinction time of the more variable species is longer than the extinction time of the population comprising less variable individuals. Also the statement formulated at the beginning of this paragraph, as individual-based models show, is not unconditional. Many depend on character of the distribution which describes species variability. Earlier papers dealing with this question applied symmetric distributions of features expressing species variability (Begon and Wall 1987). However, these authors stressed also that an asymmetric distribution should be more appropriate in this case. Indeed, individual-based models which I presented in this paper proved that the meanings of the terms competitive exclusion and coexistence of competing species depend on the shape of the distribution describing species variability. It is important whether this distribution is symmetric or asymmetric. Please note also that individual-based approach to this question enables us to formulate precisely what does species variability means. In the classical models, the distribution of a parameter describing the interspecific competitive ability of species which is rather hard to measure, was used. In the individual-based models, it is the distribution of individual traits connected with the assimilation of resources which are subject to competition.

When the distribution describing species variability is symmetric, all statements about competitive exclusion and coexistence of competing species are of a statistical nature. When properly formulated, the principle of competitive exclusion should be put like this: if competition episodes between two species repeat many times, then the longer extinction time of the species with a higher individual variability will be more frequent than that of the species with a lower individual variability. On the other hand, the coexistence of two competing species with identical individual variability means that these populations will have longer extinction times with equal frequency.

This situation arises from the character of the distribution of individual traits. They were drawn from a normal, thus symmetric, distribution. An increase in the variance of this distribution means that in the population appeared individuals whose presence increased population persistence, along with individuals less capable of reproduction, thus decreasing population persistence. Drawing individual traits at each generation, especially when the population is small, can lead to the situation that even less valuable individuals can appear in populations with higher individual variability. However, with appropriately large differences in individual variability between two populations, the advantage due to drawing individuals with traits enhancing long extinction times of the more variable population can be so large that the competitive exclusion of a more variable species by a less variable species will be much less frequently observed. For the same reason, we will observe the coexistence (in the above statistical sense) of the two species even when they differ in their individual variabilities, but on the condition that these variabilities are great enough (but not much different) to enhance the long extinction times of each of them.

Different situation arises when the distribution illustrating species variability is asymmetric, because now the increase of variability can go through an increase in the number of individuals with traits enhancing or weakening the long extinction times of the more variable population. For the case of a single population, this favorable effect of individual variability does not depend on how we will attain this variability of the population—whether through an increase in the number of weak individuals or strong individuals. It is only important that they differ among themselves. However, for two competing populations, the situation looks a little different.

Hierarchy of individuals, the amount of food that each of them will obtain, with all further consequences of this resource partitioning resulting from intraspecific competition, is now dependent on each of the two populations separately. However, individuals of both species use the common food. Thus, individuals of both populations will experience the maximum level of food at the same time, and later the minimum also at the same time.

The use of common resources by individuals of both species strongly synchronizes their dynamics: their numbers will attain a minimum simultaneously. Results of interspecific competition depends on which individuals will be present at a minimum in each of these populations.

If an increase in population variability is due to an increase in the contribution of valuable individuals (*s*_*min*_^1^ = *s*_*min*_^2^ and *s*_*max*_^1^ ≠ *s*_*max*_^2^)—consuming more food during interspecific competition, with larger body weights, and higher juvenile production—then their importance for population persistence is so great that the more variable species will be characterized by a higher persistence, that is, it will have a longer time to extinction. Just the presence of these individuals (when one population is more variable) or their absence (when the other population is less variable), will determine the fate of competing populations at the minima of their numbers.

However, if an increase in the population variability relies on an increase in the contribution of lower quality individuals (*s*_*max*_^1^ = *s*_*max*_^2^ and *s*_*min*_^1^ ≠ *s*_*min*_^2^), which gain less food, attain less body weight, and thus produce less juveniles, then the resulting differences in the sizes of these populations will start playing an important role. The more variable population is enriched in individuals dying without producing offspring. In a sense, this corresponds to an increased mortality, and causes that the more variable population will be characterized by a smaller size. This limits the chance that at the minimum there appears an individual capable of producing juveniles under these conditions. Therefore, the more variable population will eventually face a greater risk of extinction at each minimum. Instead, the less variable population will not lose its potential variability for the production of less valuable individuals and, as a result, it will increase. This increases the chance that at minimum numbers there will appear an individual capable of reproduction.

Moreover, if we have a look at Fig. 18B, we will see that at the differences in individual variability of that kind, a downward shift of the left end, illustrating the partitioning of resources among individuals of the more variable population, will lead to a simultaneous slight reduction of assimilation by heavy individuals. This additionally reduces the chance to persist with the minimum numbers.

The analysis of the model in which individual variability is a result of intraspecific competition indicates large ranges of parameter values enabling the long-lasting coexistence of two competing species and their simultaneous extinction. First of all, this concerns the situation when, independent of the way of incorporation of individual variability of the two species, they are equally variable. Besides, the coexistence of two species is also possible when these species differ in their individual variability, but on the condition that the values of the model parameters responsible for this promote long extinction times (both species should be sufficiently variable). This is the case in the lower-left part of Fig. 19 and in the upper-right part of Fig. 26.

In all cases of species coexistence or competitive exclusion of species, the system consisting of two populations with asymmetric distributions describing the variability of them behaves in a purely deterministic way. If there is coexistence of species, both of them go extinct at the same time. When the more variable species excludes the less variable one, it happens always. Not more often than opposite outcome of competition as it was observed in case of symmetric distributions.

At this point, it is worth remembering another peculiar property of the population dynamics described by this model. Each population will die sooner or later, although for certain values of the model parameters, the extinction times can be very long (exceeding 1000 time steps, or over 1000 generations, which is a lot for a local population with nonoverlapping generations). Thus, in the situation when both populations go extinct simultaneously, we can say of a long coexistence of two species only when their extinction times are long. However, a large area of coexistence and simultaneous extinction of the two competing species is in the upper-right part of Fig. 19 and near the right edge or the upper edge of it, that is, when the species differ in individual variability and the differences are even big but the variability of each of them is small. In this case, we observe a relatively short coexistence of both competitors and a simultaneously but relatively quick extinction of both of them. It is difficult to speak of the coexistence of species. In this case, we rather have to do with a simultaneous demographic catastrophe of both species in a short time.

In both models presented in this paper, there are no direct interactions between individuals of different species. Interactions between individuals, which are assumed in the models, are confined to each species separately. Intraspecific competition for resources produces individual variability within each population. Interspecific interactions operate through the use of common resources. The resource conditions of each species changes because the other species uses the same resources. This is a very simple (maybe the simplest) way of introducing interspecific competition into an individual-based model of this kind. In classical models, the situation is different. There are specific parameters which are responsible for interspecific competition in classical models. Their values can be different for different species, indicating that they differently react to competition from the side of other species. There is no easy way to introduce such parameters into individual-based models presented in this paper. Let us notice, however, that the global competition is assumed in these models. This together with the assumption about the use of common resources probably means that both species can’t be very different in the average values of model parameters. Maybe the only difference between them can be expressed in the variability of these parameters. The situation is much more simpler for local competition, which we can assume in the case of sedentary organisms (for instance terrestrial plants). If interactions occur in pairs of individuals and their strength depends, for instance, on the size of neighbour and the distance to it, we can very easily differentiate the intensity of interactions between individuals of the same and different species for instance in the way proposed by Wszomirski et al. (1999).
